# Patient experiences of acute medical same day emergency care: a mixed-methods cross-sectional survey across two hospitals in England

**DOI:** 10.1136/bmjopen-2026-124245

**Published:** 2026-10-22

**Authors:** Sue Dean, Holly Blake, Julian Barratt, Jo Leonardi-Bee

**Affiliations:** 1 Same Day Emergency Care, United Lincolnshire Hospitals NHS Trust, Boston, UK; 2 School of Health Sciences, University of Nottingham, Nottingham, UK; 3 NIHR Biomedical Research Centre: Nottingham, Nottingham University Hospitals NHS Trust, Nottingham, UK; 4 Aston Medical School, Aston University, Birmingham, UK; 5 Centre for Evidence Based Healthcare, School of Medicine, University of Nottingham, Nottingham, UK

**Keywords:** Emergency Service, Hospital, GENERAL MEDICINE (see Internal Medicine), Patient-Centered Care, Patient Satisfaction, Quality of Health Care, Surveys and Questionnaires

## Abstract

**Abstract:**

**Objectives:**

To evaluate patient experience in same day emergency care (SDEC) across two sites in England and examine variation in experience by age, gender, ethnicity and disability.

**Design:**

Cross-sectional survey with embedded free-text content analysis.

**Setting:**

Two acute medical SDEC departments in England.

**Participants:**

Adults (aged ≥18 years) attending SDEC between October 2024 and June 2025.

**Primary and secondary outcome measures:**

Primary outcome: overall patient experience and experience across eight SDEC journey phases. Secondary outcomes: subgroup differences by demographic characteristics and recurring patterns identified through content analysis of free-text comments.

**Results:**

There were 312 respondents (response rate 78%); demographic characteristics were broadly similar across sites. Overall experience was highly rated (median 10, IQR 8–10; maximum score=10). Across the eight journey phases, item-level responses and composite scores were strongly positive, with most domains clustered at the upper end of the scale. The Waiting domain showed more mixed responses, and the Leaving SDEC domain displayed a wider spread of scores. Qualitative content analysis identified recurring positive patterns related to coordinated care, interpersonal communication and environmental comfort

Subgroup analyses showed no significant differences by site or gender. Respondents aged >75 years had higher odds of reporting a positive experience in the Waiting domain, respondents from Other ethnicities (all non-white-British groups) had lower odds of reporting positive experiences and disabled respondents reported lower odds of positive experiences in the Doctors and Nurses domain.

**Conclusions:**

SDEC patients reported consistently positive experiences, with only a few areas showing more mixed views. Most demographic groups reported similar experiences, but lower ratings among respondents from Other ethnicities and disabled patients highlighted equity gaps. Although SDEC is primarily established in England, similar models are emerging internationally and these findings offer insights for acute care pathways globally.

**Trial registration number:**

Open Science Framework, https://doi.org/10.17605/OSF.IO/2CB6K.

**STRENGTHS AND LIMITATIONS OF THIS STUDY**

- A validated patient-experience survey adapted from established emergency care measures provided a structured and reliable assessment of same day emergency care (SDEC) experience.
- The large sample size and high response rate (78%) provide a robust dataset, although the true denominator of patients offered the survey is unknown, limiting assessment of non-response bias.
- Conducting the survey across two SDEC sites enhances ecological validity, but single-region sampling may limit generalisability.
- Free-text comments enriched interpretation of quantitative findings, though many were brief and offered limited qualitative depth.
- Paper-based distribution and completion during or immediately after care may have influenced accessibility and introduced social desirability or fatigue effects.

## Introduction

Same day emergency care (SDEC) services are designed to provide timely assessment, treatment and discharge for patients presenting with acute medical conditions, without requiring overnight admission.^1^ In the acute medical context, SDEC typically manages adults with undifferentiated medical problems who are clinically stable enough to be assessed, investigated and treated within a same-day episode of care. Patients are streamed from the emergency department (ED), general practice, ambulance services or National Health Service (NHS) 111 into a dedicated assessment area where senior decision-makers have access to diagnostics and short-term treatments. SDEC has been operational for a relatively short period of time, building on previous ambulatory care models and has been rolled out throughout England in a top down policy driven approach,^2^ across a range of specialities, with a limited evidence base.^3^

SDEC services are considered essential to the future provision of acute medical care, in an ageing society with limited healthcare resources and are conjointly endorsed by the Royal College of Emergency Medicine and the Society for Acute Medicine.^4–6^ The focus of this research was on acute medical SDEC, as medical patients represent a significant proportion of emergency admissions in the NHS. Typically, up to 30% of emergency admissions are discharged from an inpatient stay within 24-hours of arrival, suggesting that admission avoidance, using SDEC services, can meet that need and reduce pressure on inpatient beds,^1 7^ as recommended in The NHS Long Term Plan.^8^

Internationally, related services exist, such as ambulatory care units, short-stay units and ED observation units, but these models typically provide bed-based observation for 24–72 hours or focus on lower-acuity presentations. In most countries, ambulatory care refers to outpatient or primary care activity rather than acute same-day emergency assessment. In contrast, the English acute medical SDEC model is designed as a non-bedded pathway with an expected stay of under 12–24 hours and is unique in being nationally mandated, standardised and implemented at scale across the NHS. Accordingly, its novelty lies not in the general concept of same-day care, which has parallels internationally, but in its specific configuration, governance and national policy status within the UK.^3^

Patient experience research is increasingly recognised as a vital component of evaluating healthcare quality as it offers direct insight into how care is delivered from the patient’s perspective, revealing gaps, strengths and opportunities that clinical or operational data cannot capture.^9–11^ Despite the rapid expansion of SDEC services across England, recent reviews highlight a notable absence of published studies exploring patient experience within this model of care.^12 13^ Internationally, directly comparable evidence is limited, as services labelled ambulatory care in many countries refer to outpatient or primary care activity rather than acute same-day emergency assessment. Existing research therefore focuses on adjacent models, such as ambulatory emergency care pathways, ED fast-track areas and rapid diagnostic clinics, which differ in acuity, purpose and operational design.

To address this evidence gap, this study aimed to assess patient reported experiences of care processes and perceived quality within acute medical SDEC units at two hospitals in England. Specifically, the study examined: (1) overall patient experience; (2) experience across eight SDEC journey phases; (3) variation in experience by age, gender, ethnicity and disability; and (4) recurring patterns in free-text comments.

## Methods

### Study design

A cross-sectional mixed methods survey was conducted to examine patient reported experiences of acute medical SDEC across two hospital sites.

The mixed methods design combined structured experience ratings with qualitative free-text responses to capture both the measurable and narrative dimensions of patient experience. Reporting of the survey component was informed by the Consensus-Based Checklist for Reporting of Survey Studies (CROSS) (online supplemental file 5).^14^

### Study setting

This study was conducted in two acute medical SDEC units within a large rural and coastal county in the East Midlands of England, specifically at Lincoln County Hospital and Pilgrim Hospital Boston (United Lincolnshire Teaching Hospitals NHS Trust). The county has a resident population of 768 400,^15^ which increases substantially during the tourist season. Both hospitals operate dedicated non-bedded acute medical SDEC services that provide same-day assessment, investigation, treatment and discharge for adults presenting with undifferentiated acute medical conditions. Patients are referred from the ED, general practice, ambulance services and NHS 111. Only patients who are ambulatory, clinically stable and who have capacity are accepted.

Each SDEC unit functions as a distinct assessment area staffed by senior clinical decision makers with access to same-day diagnostics and short-term treatments. Both services operate 7 days a week and manage patients expected to complete their episode of care within the 14 hours per day opening time, although patients may return on subsequent days for further review or treatment. The two units differ in size and throughput, reflecting local population needs, but each provides a pathway intended to reduce avoidable overnight admissions and support acute medical flow within the wider hospital system.

### Sampling and recruitment

Survey participants were adult (≥18 years) patients attending SDEC between October 2024 and June 2025, recruited through convenience sampling. Eligible participants were those who were clinically stable, able to provide informed consent and sufficiently well to complete a short paper survey during their SDEC visit. To support inclusion, SDEC staff were asked to offer the survey to a wide range of patients, including those from diverse demographic backgrounds and individuals who might require additional assistance to participate. Assistance was limited to practical support such as reading questions aloud or helping patients handle the paper survey; staff did not provide interpretive guidance or influence responses. Translation support was available for patients who required help understanding the survey content. Surveys were distributed across two contrasting hospital sites, one urban and one rural/coastal, to capture variation in service context and enhance the relevance of the findings across different SDEC settings.

Each survey pack included a participant information sheet outlining the study purpose, contact details and consent process. Staff received protocol training and were available to answer questions. Participation was voluntary, and completion and return of the survey constituted implied consent, consistent with accepted practice in hospital-based survey research.^16^

A target of 400 distributed surveys (200 per site) was set to achieve a 95% CI with a ±5% margin of error for estimating the proportion of respondents, assuming a conservative 50% response rate.^17^

### Data collection

The study used the 2012 Accident and Emergency Department Questionnaire (AEDQ), a freely available Patient-Reported Experience Measure developed by the Picker Institute, which has demonstrated patient-level reliability, construct validity and discriminative capacity.^18–20^ Minor wording changes were made to reflect the SDEC context, replacing ‘Accident & Emergency’ with ‘Same Day Emergency Care’ and adding ‘advanced practitioner’ where relevant to align with local staffing (online supplemental file 1). Pilot testing was not undertaken because the AEDQ has been widely used in emergency care settings since 2003, with multiple validated iterations.^18 21^ More recent versions of the survey, held under Care Quality Commission copyright, were unavailable despite repeated permission requests, so the 2012 version was used.

The survey was administered in paper format and took approximately 8–12 min to complete. Completed surveys were placed in a collection box at the nurse’s station.

The final instrument contained 52 items across 11 domains. The first eight domains corresponded to stages of the patient journey, capturing procedural and interpersonal aspects of care: Arrival (first impressions and reception), Waiting (waiting times and communication about delays), Doctors and Nurses (communication, professionalism and confidence in staff), Care and Treatment (clarity, involvement and overall clinical care), Tests (experiences and explanations relating to investigations), Pain (assessment and management), Environment and Facilities (cleanliness, comfort and privacy) and Leaving the Department (discharge information and understanding of next steps). The Overall Experience domain included privacy and dignity and overall impressions. The maximum possible score for each question was 10. The final section invited free-text comments to complement the structured experience ratings, with the questions: ‘Was there anything particularly good about your visit to the SDEC department?’, ‘Was there anything that could have been improved?’ and ‘Any other comments?’

The quantitative survey dataset and anonymised free-text responses generated for this study have been deposited in the University of Nottingham Research Repository.^22^

### Data analysis

Survey question responses and free text comments were entered into Microsoft Excel. Surveys with fewer than five completed items were excluded, and item-level response counts were reported to indicate the number of participants contributing data to each question; percentages were calculated using these denominators.

A patient journey approach guided the quantitative analysis, reflecting the conceptual structure of the AEDQ. Evaluative items were converted to 0–10 scores,^23^ and composite scores for each journey domain were calculated by averaging non-missing evaluative items. Because several domains showed skewed distributions and ceiling effects, both mean (SD) and median (IQR) were reported to provide robust and comparable summary statistics. Item-level response distributions and composite score calculations for all survey domains were produced as part of the descriptive analysis (online supplemental file 2). Descriptive statistics were generated for demographic variables. All composite domains were non-normally distributed (Shapiro-Wilk p<0.05), so subgroup analyses used dichotomised scores (positive ≥8 vs non-positive <8). Associations between categorical variables were examined using χ² or Fisher’s exact tests, with crude ORs calculated and the Haldane correction applied where zero cells occurred. Logistic regression was used to estimate adjusted ORs, controlling for site, age group, gender, ethnicity and disability; crude estimates were retained when sparse data prevented adjustment. Model fit was assessed using the Hosmer-Lemeshow test, and logistic regression assumptions were checked. Site differences were further explored by comparing crude and adjusted ORs to assess robustness. Effect sizes were quantified using Cohen’s h (0.2 small, 0.5 moderate, 0.8 large). Statistical significance was set at p<0.05. All statistical analyses were conducted in R via the R Studio interface.

Gender was coded as Female, Male and Other; the ‘Other’ category was retained for descriptive reporting but excluded from inferential analyses due to sparse data. Ethnicity was coded as white British versus Other, as small numbers across minority groups produced sparse cells that prevented stable estimation in subgroup analyses. Disability was coded as Yes or No, with ‘Prefer not to say’ responses excluded from inferential analyses because of low counts.

Free-text comments were analysed using qualitative content analysis appropriate for brief survey responses. A framework informed by the survey’s conceptual structure (the patient-journey domains) guided initial coding in NVivo (V.15). Categories were refined inductively through close reading of the data and grouped into descriptive categories aligned with each stage of the patient journey.

Qualitative and quantitative analyses were undertaken independently, with integration occurring during the synthesis and presentation of results to provide a coherent mixed-methods interpretation.

The lead researcher is a PhD candidate and advanced nurse practitioner working within SDEC, bringing detailed contextual knowledge of the pathway and its operational environment. This insider perspective informed the development of the study and the interpretation of findings, while also requiring reflexive attention to potential assumptions arising from clinical experience. Academic supervisors with expertise in survey methodology and qualitative analysis, and who were independent of the participating SDEC services, provided oversight and contributed to analytical decisions. A patient and public involvement (PPIE) representative also reviewed the qualitative interpretation to ensure that patient perspectives were appropriately reflected and to support transparency in the analytical process.

### Patient and public involvement

Public and patient involvement informed the study design through consultation with the United Lincolnshire Teaching Hospitals NHS Trust PPIE Group and the NHS England Experience of Care SDEC Quality Group. A subgroup of four PPIE members contributed to the development of the study proposal, participant information materials and consent documentation. A PPIE representative provided feedback on the qualitative findings to ensure that patient perspectives were appropriately reflected.

## Results

### Participant characteristics

Surveys were distributed across two SDEC sites, Lincoln and Boston Hospitals, with 200 questionnaires issued at each site (n=400). A total of 314 responses were returned (78% response rate): 136 from Lincoln (68%) and 176 from Boston (88%). Two surveys were excluded due to insufficient completion, resulting in an analytical sample of 312 participants (figure 1).

**Figure 1:**
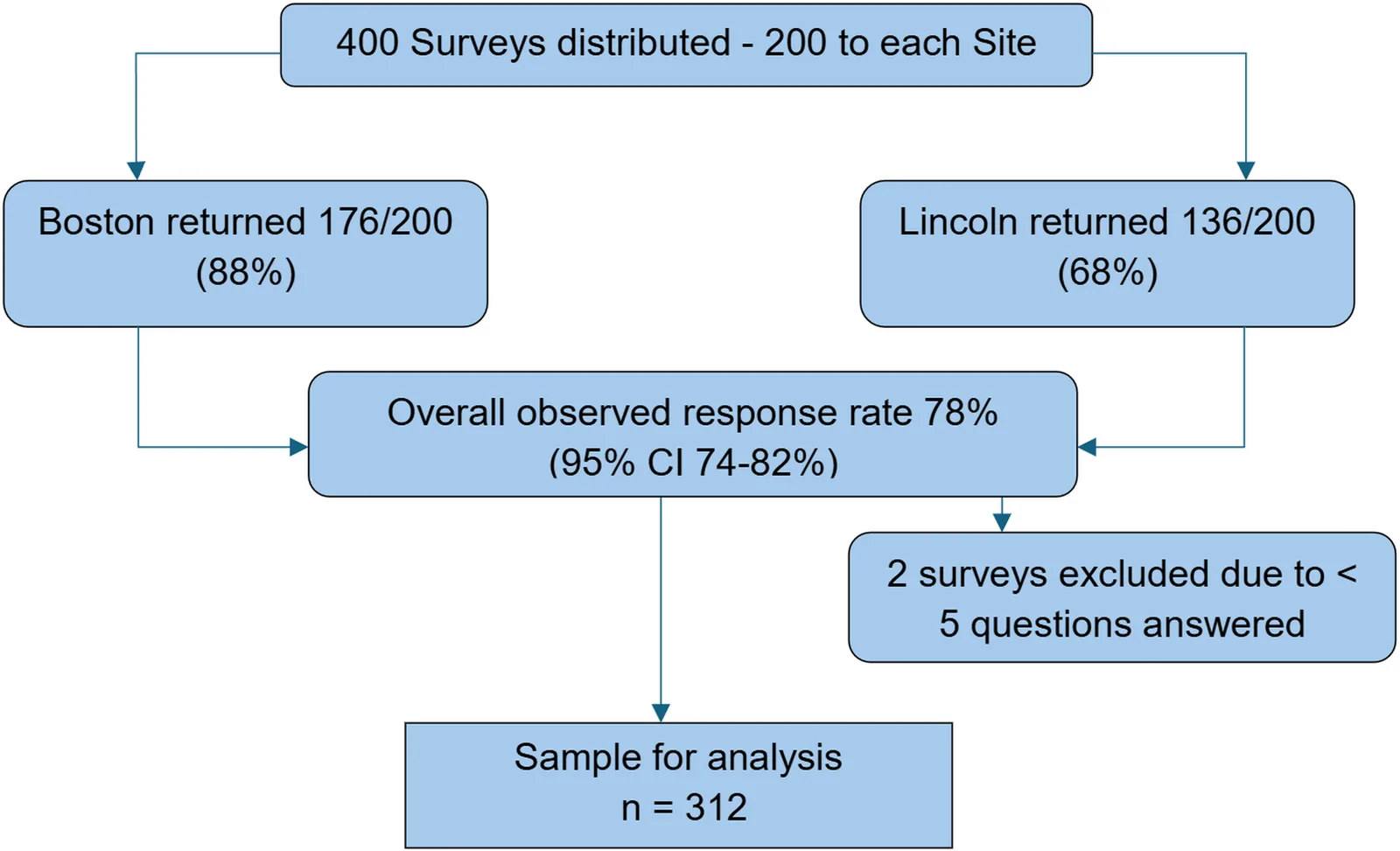
Survey distribution, response rates and participant inclusion.

Respondents had a median age of 60 years (IQR 40–73; n = 257; table 1), with 56.6% identifying as female and 42.6% as male. Most participants identified as heterosexual (92.0%, n = 230) and white British (90.0%, n = 244). Just over half reported a religious affiliation, most commonly Christian (58.5%, n = 148). Disability was reported by 45.4% of respondents (n = 108), and among these, 73.6% indicated that it affected their daily life.

**Table 1:** Demographic data of survey respondents

Variable	Category	N (%)	Missing N
Age	Median (IQR)	60 (33)	55
Gender	Female	146 (56.6)	54
	Male	110 (42.6)	
	Other	2 (0.8)	
Sexual preference	Bisexual	7 (2.8)	62
	Gay/lesbian	7 (2.8)	
	Heterosexual	230 (92)	
	Other	6 (2.4)	
Religion	Christian	148 (58.5)	59
	Muslim	6 (2.4)	
	No religion	95 (37.5)	
	Prefer not to say	4 (1.6)	
Ethnicity	African	4 (1.5)	41
	Any other Asian background	1 (0.4)	
	Any other Mixed ethnic background	1 (0.4)	
	Any other white background	13 (4.8)	
	Bangladeshi	1 (0.4)	
	English/Welsh/Scottish/ Northern Irish/British	244 (90)	
	Gypsy or Irish Traveller	1 (0.4)	
	White and black African	3 (1.1)	
	White and black Caribbean	3 (1.1)	
Disability	No	123 (51.7)	74
	Prefer not to say	7 (2.9)	
	Yes	108 (45.4)	
Disability impact	No	28 (26.4)	2
	Yes	78 (73.6)	
Site (312)	Boston	176 (56.4)	0
	Lincoln	136 (43.6)	

### Overview of patient reported experience

Results are presented across the nine patient journey domains: Arrival (first impressions and reception), Waiting (waiting times and communication about delays), Doctors and Nurses (communication, professionalism and confidence in staff), Care and Treatment (clarity, involvement and overall clinical care), Tests (experiences and explanations relating to investigations), Pain (assessment and management), Environment and Facilities (cleanliness, comfort and privacy), Leaving the Department (discharge information and understanding of next steps) and Overall Experience.

Composite scores were generated for each domain to summarise experience; all scores were on a 0–10 scale. (table 2). Arrival was rated positively overall (mean 8.93), though with a wider spread than other highly rated domains. Environment, Care and Treatment, and Doctors and Nurses received the highest ratings (means 9.46, 9.42 and 9.30, respectively), with narrow IQRs and tightly clustered distributions near 10 (figure 2), indicating consistently positive experiences. Tests also showed generally favourable ratings (mean 8.63) but with a slightly broader distribution. In contrast, Waiting (mean 7.22, SD 2.51) and Leaving SDEC (mean 7.96, SD 2.91) demonstrated lower mean scores and greater variability, reflecting more mixed experiences. The Pain domain displayed a bimodal distribution, with responses clustering at both extremes of the scale. Overall Experience remained positively skewed, with most respondents rating their care near the upper end of the scale. Response counts varied across domains, with lower completion for Pain (as only respondents who experienced pain were asked to rate this domain) and for Leaving SDEC, where some participants chose not to complete the final section of the survey after being informed they could leave the unit.

**Table 2:** Summary statistics for composite scores across survey domains

Domain	N	Mean	SD	Median	IQR
Arrival	274	8.93	2.34	10.00	0.00
Waiting	311	7.22	2.51	7.67	3.66
Doctors and Nurses	295	9.30	1.38	10.00	0.92
Care and Treatment	286	9.42	1.18	10.00	1.00
Tests	268	8.63	2.50	10.00	1.67
Pain	103	8.83	2.45	10.00	0.00
Environment	287	9.46	1.16	10.00	0.82
Leaving SDEC	235	7.96	2.91	10.00	3.00
Overall Experience	260	9.00	1.72	10.00	1.00

Composite scores were calculated as the mean of valid item responses per domain, excluding missing values. The maximum possible score for each was 10. Participation counts (n) reflect the number of respondents with at least one valid item in the respective domain.

SDEC, same day emergency care.

**Figure 2:**
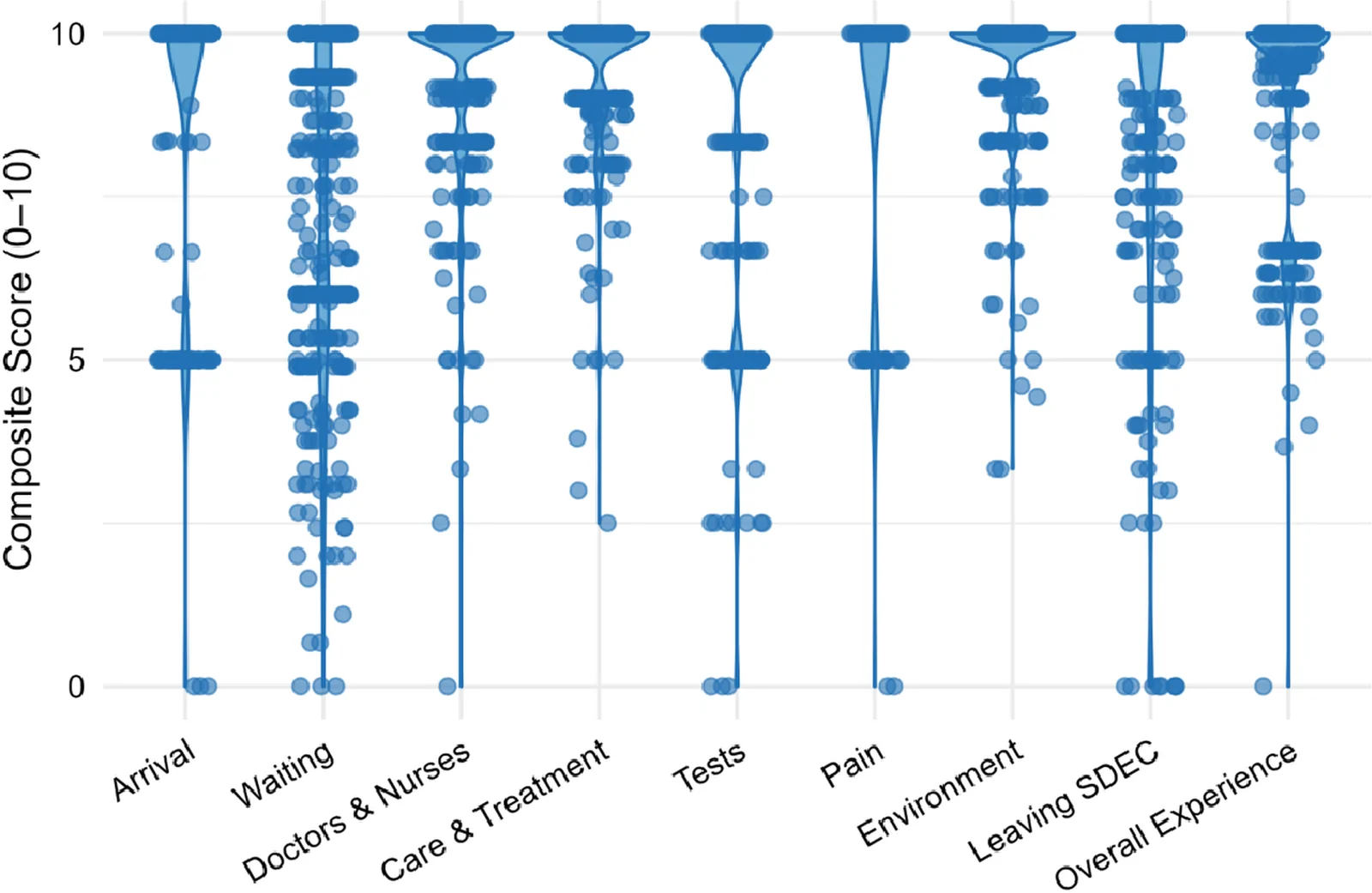
Distribution of composite scores across survey domains. Violin plots display the distribution and density of composite scores (range 0–10) for each patient journey domain. Individual respondent scores are shown as jittered points. Composite scores represent the mean of valid item responses within each domain, with missing values excluded. SDEC, same day emergency care.

In addition to quantitative ratings, 127 respondents (41%) provided free-text comments. These were typically brief (median 12 words; range 2–112), supporting the use of content analysis focused on recurring issues rather than thematic saturation. The qualitative findings are presented below across the patient-journey domains.

### Patient journey domains

#### Arrival

Arrival-related items provided insight into how patients entered the service and their first impressions of SDEC. Most participants reported arriving without prior contact, with general practitioner (GP) surgeries and the ED being the most common referral sources; only a small proportion arrived by ambulance. Item-level responses indicated consistently positive experiences of coordination, handover and reception processes, despite variation in referral pathways.

Free-text responses relating to the Arrival phase were limited, but they offered insight into early impressions of the service. Comments touched on initial delays, first interactions with staff and the clarity of information provided on arrival. Some respondents highlighted smooth coordination and relief at avoiding long waits elsewhere, for example: “I am so pleased I went to my GP and came [direct to SDEC] I did not wish to sit in A&E [accident and emergency] for 30 hours plus” (P283). Others emphasised positive interpersonal encounters, noting a “Fantastic meet and greet” (P136) and that “From the minute we arrived… I was seen very quickly and everyone treated me with care and kindness” (P292). A few comments pointed to gaps in orientation, such as wanting clearer information on arrival: “On arrival just would be good to know straight away the simple things like drinks etc.” (P285). Overall, while the Arrival phase did not generate extensive feedback, it captured important first impressions of coordination, welcome and basic information needs.

#### Waiting

Waiting-related survey items captured patients’ experiences of time to initial contact, examination, information about delays and overall visit duration. Respondents generally reported short waits to speak to a clinician and to be examined, although ratings for information about waiting times were more variable. The composite waiting score reflected this mix of prompt clinical contact alongside less consistent communication.

Free-text comments echoed these patterns. Several respondents described long or uncertain waits, noting that “They should work on the waiting time” (P43, P264) and “The wait for scans was a long time” (P86). Others highlighted a lack of clarity about what they were waiting for: “I had no idea what I was waiting for” (P301) and “It was just a long wait so giving people an idea of this so they can bring books or snacks would be helpful” (P62).

At the same time, many comments emphasised the positive role of staff during this phase. Patients described supportive interactions that helped mitigate delays, such as “A lovely wait with lovely staff” (P235). A few respondents also expressed overall satisfaction with the pace of their visit, noting that “I was finished quicker than I thought” (P48) and simply “quick” (P156).

The qualitative feedback reinforces the quantitative findings: waiting times varied, and uncertainty was a source of frustration for some, but staff interactions often shaped the waiting experience positively.

#### Doctors and Nurses

Staff related survey items captured patients’ experiences of communication, attentiveness, emotional support and opportunities for discussion. Scores were consistently high across these domains, with strong ratings for time to talk, clarity of explanations, feeling listened to and trust in staff. The composite score reflected this pattern, with most respondents reporting very positive experiences.

Free-text comments reinforced these findings. Many respondents described staff as kind, professional and attentive, for example: “Staff were all lovely and very friendly” (P3), “Very professional & courteous staff who were able to take time for you” (P14) and “I would like to say it is the best department that I have visited for politeness, care and attention and treatment, lovely staff” (P30). Others highlighted feeling listened to and cared for, noting “Excellent care, friendly and caring staff, attentive, felt I was treated as an individual and not just a number” (P58) and “The kindness and understanding of all staff, I feel privileged to be cared for by such amazing NHS staff” (P256). Team-based care was also praised, such as “The HCSW [health care support worker]… was absolutely fantastic… all staff friendly and very helpful, fantastic team” (P103).

A smaller number of comments pointed to communication challenges or concerns about privacy. One respondent described an interaction that felt abrupt: “I had no idea what I was waiting for… the healthcare assistant snapped across the room… she continued to be rude to me” (P301). Others mentioned confidentiality issues, such as “Confidentiality - nurses talking at the desk so all can hear is not great” (P237) and “The reading out of personal information… in an open space” (P121). Overall satisfaction with clinical staff was also evident, for example: “I was extremely happy with doctors and nurses all round, felt respected” (P170).

The qualitative feedback strongly aligns with the quantitative results: interpersonal care, professionalism and clear communication were central to positive experiences with doctors and nurses, with only occasional concerns about communication style or privacy.

#### Care and Treatment

Survey items relating to care and treatment captured patients’ experiences of information provision, privacy, access to help, consistency of communication and involvement in decisions. Scores were consistently high across these domains, with strong ratings for information about treatment, privacy during examination and access to help when needed. Involvement in decision-making was also rated positively.

Free-text comments reinforced these findings. Many respondents described compassionate and attentive care, often highlighting the emotional support provided by staff. Examples included: “I was upset and anxious about my visit so they allowed my family to stay with me” (P129), “The way the staff cared for me… made me a much-needed cuppa and personally rang my wife… to let her know what is happening” (P148) and “It was a calmer, quieter environment [than ED]… all the staff went over and above to help” (P281). Patients who had attended SDEC regularly also described consistently positive experiences, such as “I always receive prompt treatment… I am always treated exceptionally well… excellent level of care” (P55).

Comments also reflected clear communication and thorough explanations, for example: “Was seen promptly… all explained their roles & what I could expect” (P93) and “The nurse and clinician were very thorough and caring and took our concerns seriously” (P287). Some respondents highlighted coordinated care, noting “Throughout the treatment has been outstanding… a fantastic service” (P168) and “Always attentive & quick to start treatment… up to date with all my personal health issues” (P218).

No negative comments were recorded regarding care or treatment delivery. In general, the qualitative feedback aligned with the quantitative findings, with patients consistently describing their care as high-quality and supportive.

#### Tests

Survey items relating to diagnostic testing captured patients’ experiences of having the purpose of tests explained, receiving results and understanding those results. Scores were consistently high across all items, with strong ratings for explanation of test purpose, receipt of results before leaving and clarity of result communication.

Free-text comments for this phase were limited but reinforced these findings. Several respondents highlighted clear explanations and supportive communication, for example: “They explained everything and answered my questions” (P21), and “Doctor… gave me clear explanations of process & timings” (P271). Others emphasised professionalism and interpersonal care, such as “All staff very professional and caring, explained everything to me” (P36).

No negative comments were recorded in this phase. Across the qualitative data, feedback aligned with the quantitative results, with patients describing diagnostic testing as well-explained, well-coordinated and delivered with professionalism and clarity.

#### Pain management

Survey responses relating to pain control were provided only by those who reported experiencing pain during their visit. Most respondents indicated that staff did everything possible to help manage their discomfort, with scores clustered at the upper end of the scale.

Free-text feedback for this phase was minimal, with only one comment identified. The respondent noted “good ability to request additional painkillers” (P46), reflecting timely access to support when needed.

The data indicate that patients who experienced pain felt well supported, with staff responding promptly and effectively to their needs.

#### Environment and Facilities

Survey items relating to the care environment captured patients’ experiences of cleanliness, safety, comfort and access to food and drink. Scores were consistently high across all items, with very limited variability. The composite score reflected this pattern, indicating strong satisfaction with the physical environment and facilities.

Free-text comments offered a broader view of these experiences. Many respondents described the environment as calm and comfortable, for example: “Comfortable at all times” (P126) and “Calm environment after A&E” (P14). Food and drink provision was frequently mentioned as a positive aspect of care, with comments such as “Food & drink is a helpful touch. A much nicer experience than A&E & other departments.” (P234) and “Continuous offering of light drinks etc.” (P299). Staff contributions to comfort were also highlighted, including “The HCA [health care assistant] got us all tea biscuits & sandwiches which was incredibly helpful” (P62).

Alongside these positive reflections, several respondents identified areas for improvement. Comments included concerns about food quality: “Sandwiches were disgusting the cheese was like rubber and tasted horrid” (P115), and cleanliness: “Toilet not clean” (P209) and “Cleanliness could be improved” (P239). Others mentioned environmental issues such as “(Improve) air conditioning” (P136) and the appearance of the department: “It looks like the department was made up of items… other departments don’t want” (P214). Some respondents also noted gaps in provision for specific needs, for example: “Availability of snacks for diabetics (could be improved)” (P107) and “No diabetic friendly food” (P280).

The qualitative feedback aligns with the quantitative findings: patients generally experienced the environment as clean, calm and supportive, though this phase generated more critical comments than others, particularly regarding food quality and aspects of the physical environment.

#### Leaving SDEC

Survey items relating to discharge captured patients’ experiences of medication explanations, follow-up information and clarity around next steps. Scores were generally high across these items and strong ratings for understanding the purpose of medication and receiving clear discharge information. Advice on resuming normal activities showed greater variability.

Free-text comments for this phase were limited but offered insight into these patterns. Some respondents described supportive interactions and coordinated discharge processes, for example: “They also went out of their way (all staff) to keep us comfortable while we waited for a bed in the hospital ward” (P287) and “I was… discharged with full explanation and treatment” (P271). Others highlighted areas for improvement, such as “Could improve information about drug prescribed” (P304). A small number of comments referenced delays or uncertainty, including “My treatment usually takes 30 mins but have to wait many hours to go home” (P57); although that comment is related to having to wait for hospital transport so outside SDEC’s control.

The qualitative feedback aligns with the quantitative findings: most patients reported clear communication and supportive staff at discharge, though a minority experienced gaps in information or delays.

#### Overall Experience

Survey items relating to overall experience captured patients’ impressions of dignity and respect, general satisfaction and awareness of how to make a report. Scores were very high for feeling treated with respect and dignity and for overall experience. Awareness of issues information showed greater variability.

Free-text comments echoed these findings. Many respondents described excellent care and high satisfaction, for example: “Excellent throughout” (P51), “Fantastic service” (P76) and “Cannot fault the service” (P94). Several highlighted the quality of interpersonal interactions, such as “All very good excellent care” (P38) and “Amazing team & service” (P218). Others commented on efficiency and smooth flow of care, noting “There was an air of efficiency and constant flow of activity and attention” (P289) and “SDEC is a great place if you need it, fast access to good healthcare” (P237).

No negative comments were recorded in the overall experience section. The predominance of comments reflecting satisfaction, interpersonal care and perceived efficiency aligns with the concentration of high composite scores, indicating strong consistency between qualitative impressions and quantitative ratings of overall experience.

These findings conclude the core analysis of patient experience across the journey domains. The next section examines demographic and site-level patterns to explore potential differences in reported care quality.

### Subgroup analyses

Demographic characteristics were broadly comparable across the two SDEC sites (online supplemental file 3). Median age was slightly higher at Boston (61 years, IQR 32) than at Lincoln (58 years, IQR 29.5). The proportion identifying as disabled was modestly higher at Boston (48.8%) than at Lincoln (41.3%). Ethnicity distributions were similar, with around 90% identifying as white British at both sites, and gender profiles were consistent, with females comprising 56–57% of respondents. These differences are descriptive only; no formal statistical testing was undertaken for baseline characteristics, as inferential comparisons between sites are presented later for each patient-experience domain. Overall, the demographic similarity supported pooled analysis, with site retained as an adjustment factor in regression models.

Full crude and adjusted logistic regression outputs for all subgroup analyses are provided in online supplemental file 4. Site-level comparisons of binary composite scores showed no statistically significant differences between Boston and Lincoln across any domain. Effect estimates were small and inconsistent in direction.

Subgroup analysis compared respondents aged ≤75 years with those aged >75 years across the composite domains. Patterns were similar for crude and adjusted models. The only statistically significant adjusted association was observed in Composite Waiting, where older respondents had higher odds of reporting a positive experience (adjusted OR 2.05, 95% CI 1.01 to 4.30, Cohen’s h = 0.37, indicating a small-to-moderate effect). Composite Environment was excluded from adjusted analyses due to sparse data.

Gender comparisons showed no statistically significant differences across any composite domain, with small, adjusted effect estimates.

Subgroup analysis comparing white British and Other ethnicity respondents showed a consistent pattern across domains, with respondents from Other ethnicities generally reporting lower odds of positive experiences. Five adjusted associations reached statistical significance: Doctors and Nurses, Care and Treatment, Tests, Environment and Overall Experience, each indicating substantially lower odds of positive scores among respondents from Other ethnicities. Effect sizes ranged from small to moderate (adjusted ORs typically around 0.2–0.7).

Crude and adjusted models showed similar patterns for subgroup analysis comparing respondents who reported a disability with those who did not. The only statistically significant adjusted association was observed in the Doctors and Nurses domain, where disabled respondents had lower odds of reporting a positive experience (Cohen’s h = 0.21, indicating a small effect).

## Discussion

This study provides the first detailed account of patient experience within acute medical SDEC, offering timely insight into a rapidly expanding model of acute care. Rather than focusing solely on domain-level scores, the findings suggest that organisational conditions, such as clear pathways, calm environments and stable teams, may influence patients’ relational, procedural, diagnostic and environmental experiences. These patterns were broadly similar across demographic groups, although respondents from Other ethnicities reported lower odds of positive experiences across several domains, and disabled patients reported poorer experiences in the Doctors and Nurses domain, suggesting potential equity gaps. Participants were predominantly older adults (median age 60 years), whose expectations and interactions with emergency care may differ from younger groups.^24 25^ Women were slightly over-represented, and most respondents identified as heterosexual, reflecting patterns seen in other patient-experience surveys and highlighting the limited visibility of minority gender and sexual identities.^26 27^ The sample was less ethnically diverse than both the national population and the respondent profile of the 2024 national ED survey, consistent with the demographic profile of Lincolnshire^15 28^ and broader evidence of minority underrepresentation in healthcare research.^29 30^ Nearly half of respondents reported a disability, many with daily functional limitations, an important factor shaping engagement with healthcare services.^31^ These demographic features help contextualise the findings and indicate where caution is needed when considering generalisability to more diverse populations.

To contextualise the SDEC findings comparisons were made with the quantitative results published from the 2024 national ED survey.^28^ Although methodological differences limit direct comparison, several contrasts were notable. SDEC patients reported smoother arrival processes, more consistent interpersonal communication, clearer diagnostic explanations and more reliable pain management than ED respondents. ED patients described greater variability in many areas, with concerns linked to delays, crowding and inconsistent communication.^28^ These differences reflect structural conditions: SDEC’s smaller, more stable teams and predictable workflow support person-centred communication and timely responsiveness,^32^ whereas ED’s high volumes, operational pressures and fragmented processes limit the time and space available for relational communication and clear explanations.^24 33 34^ Environmental experience followed the same pattern, with SDEC patients reporting consistently positive perceptions of cleanliness, safety and comfort, while ED respondents described challenges associated with noise, crowding and limited access to basic facilities,^28^ consistent with the wider ED literature.^35–37^ Discharge communication in SDEC was generally positive but more variable than other domains, particularly regarding medication side effects, resuming normal activities and consideration of home circumstances. ED respondents reported even greater inconsistency, with fewer receiving written information or explanations of side effects.^28^ These patterns may reflect local contextual factors such as time pressures, competing demands and staff-to-patient ratios, which can affect clinicians’ capacity to deliver personalised discharge information.^38 39^ Resources may also be a factor, written information can support comprehension,^40^ but must be accessible for patients with lower health literacy or limited English proficiency.^41–43^ These contrasts highlight how organisational scale and workflow predictably, not simply clinical processes, shape the relational and informational quality of urgent care.

The qualitative content analysis reinforced the quantitative findings. Across the patient-journey domains, several recurring patterns characterised positive patient experience: coordinated care, interpersonal communication and environmental comfort. In contrast, uncertainty during waiting and variability in discharge information emerged as the main sources of frustration. Rather than simply echoing the domain-level patterns, these cross-domain insights illustrate how patients interpreted the organisational and relational features of SDEC, particularly the value placed on clarity, continuity and a calm environment. To avoid repetition, only a brief contrast with ED is noted here: ED free-text comments showed similar concerns around communication and waiting, but were more frequently negative, particularly regarding delays, pain management, environmental discomfort and privacy and dignity.^44^

Subgroup analyses revealed several meaningful differences. Respondents identifying as ‘Other’ ethnicities reported significantly poorer experiences across multiple domains, with small to moderate effect sizes, suggesting structural and cultural mechanisms such as reduced cultural safety or communication barriers.^45–47^ These disparities were not detected in the ED survey,^28^ likely because crowding and acuity in ED mask relational differences that become more visible in SDEC’s more interpersonal environment. Older adults reported significantly more positive experiences in the Waiting domain, likely reflecting differing expectations rather than differences in care.^24 25^ Disabled respondents reported significantly poorer experiences in the Doctors and Nurses domain, although with a small effect size, consistent with evidence that disability-related barriers often manifest in relational aspects of care.^31 48^ The free-text comments did not provide subgroup-specific explanations for these differences, likely reflecting the brevity of responses. Gender differences were minimal, and no significant variation was observed between sites. These patterns show that even within a generally positive model, interpersonal and structural inequities continue to shape how different groups experience SDEC, illustrating the need for culturally responsive, accessible and relationally inclusive practice. The contrast with ED, where overall experience ratings were markedly lower, is consistent with work suggesting that organisational design may influence relational quality and perceived dignity in urgent care settings.^28 44^ This pattern aligns with evidence that predictable pathways and calmer environments support more consistent communication and patient-centred interactions.^33 37 49 50^

Interpreting these findings requires consideration of several limitations. The true denominator of patients offered the survey was unknown because distribution was managed locally, preventing assessment of non-response bias or whether groups such as people with lower health literacy or limited English proficiency were under-represented,^51 52^ although the achieved response rate was high and compares favourably with hospital and national surveys.^28 53 54^ Free-text comments were often brief, limiting qualitative depth and paper-based distribution without a digital option may have affected accessibility or introduced social desirability or fatigue effects.^52 55^ The study was conducted across two sites within a single region, and response rates differed between them, likely reflecting contextual differences in research culture and staff engagement,^56 57^ which may limit generalisability. Interpretation of the ‘leaving’ domain may also be incomplete, as patients may only recognise gaps in discharge information once they return home and resume usual activities. Comparisons with national ED survey data should be interpreted cautiously due to differences in design and sampling. Finally, the researcher’s insider position may have influenced interpretation, although reflexive practice, supervisory discussion and PPIE involvement were used to enhance transparency and credibility.

Overall, the findings suggest that SDEC’s organisational features, such as clearer pathways, calmer environments and more stable teams, may help support patients’ relational, procedural, diagnostic and environmental experiences. Comparisons with ED are consistent with wider evidence that organisational scale, workflow predictability and team continuity may influence communication, responsiveness and the overall care environment. Subgroup patterns indicate that interpersonal and structural inequities continue to affect how different groups experience SDEC, even within a generally positive model. Together, these insights suggest that patient experience in SDEC may be influenced not only by clinical processes but also by organisational context and wider system pressures within urgent and emergency care.

These findings have practical implications for SDEC service design. Improving communication during waiting, particularly around expected timelines and next steps, may help reduce uncertainty and strengthen patient confidence. Providing more consistent discharge information, including clear written summaries and accessible explanations of medication side effects and follow-up actions, could reduce the variability observed in the Leaving SDEC domain. Addressing the disparities experienced by minority ethnic and disabled patients will require culturally responsive communication, improved accessibility and more relationally inclusive practice. These represent actionable opportunities to enhance the consistency, equity and person-centredness of SDEC care.

Future research should explore patient experience in SDEC across more diverse populations and organisational contexts, including urban settings and regions with greater ethnic diversity. Qualitative work with minority ethnic and disabled patients could help highlight the mechanisms underlying the disparities observed in this study. Comparative studies examining how different SDEC models, staffing configurations and workflow designs influence experience would also be valuable. As SDEC continues to expand internationally, cross-country evaluations could clarify which organisational features most consistently support equitable, person-centred acute care.

## Conclusion

This study provides the first detailed account of patient experience within acute medical SDEC and contributes to emerging evidence that organisational design may play a role in supporting high-quality, person-centred urgent care. The findings highlight the relational and structural mechanisms that appear to affect patient experience, including the importance of coordinated pathways, stable teams and a calm environment. Comparisons with the 2024 national ED survey are consistent with broader evidence that differences in scale, workflow and team continuity may be associated with communication and environmental quality across urgent care settings. The subgroup patterns also emphasise the need for culturally responsive and accessible practice to ensure that SDEC benefits are equitably experienced.

While SDEC is primarily embedded within the NHS in England, the pressures it seeks to address, delays and avoidable admissions, are shared internationally. The principles underpinning SDEC, such as streamlined pathways and coordinated multidisciplinary assessment, may therefore hold relevance for health systems exploring alternatives to traditional emergency care and inpatient admission. These findings offer an early evidence base to inform the development, refinement and equitable delivery of SDEC services in England and provide insights that may be informative for similar emerging models elsewhere.

## Supplementary material

10.1136/bmjopen-2026-124245 online supplemental file 1

10.1136/bmjopen-2026-124245 online supplemental file 2

10.1136/bmjopen-2026-124245 online supplemental file 3

10.1136/bmjopen-2026-124245 online supplemental file 4

10.1136/bmjopen-2026-124245 online supplemental file 5

## Data Availability

Data are available in a public, open access repository.
