# Supplementary material for: Patient experiences of acute medical same day emergency care: a mixed-methods cross-sectional survey across two hospitals in England
Source: BMJ Open. 2026 Oct 22;16(10):e124245. doi: 10.1136/bmjopen-2026-124245 (PMC13691836; doi:10.1136/bmjopen-2026-124245)
Supplement: Supplementary file 1 [file bmjopen-16-10-s002.pdf]

Additional File 2: Summary Statistics & Composite Scores by Domain

Arrival Domain (Q1-Q6) & Composite Score

Question	Category	n	Percent	Mean	SD	Median	IQR
Q1	Don't Know	11	3.6				
Q1	No	236	76.4				
Q1	Yes, between one week and one month ago	11	3.6				
Q1	Yes, more than a month ago	23	7.4				
Q1	Yes, within the previous week	28	9.1				
Q2	Ambulance service	20	6.6				
Q2	Emergency Department	133	43.9				
Q2	GP Out of Hours	7	2.3				
Q2	GP surgery	77	25.4				
Q2	Other health professional	26	8.6				
Q2	Someone else (friend, relative, colleague)	13	4.3				
Q2	Walk-in center or minor injury unit	27	8.9				
Q3	Travelled by ambulance	45	14.5				
Q4	Did staff work well together	45		9.93	0.49	10	0
Q5	Wait for handover to SDEC staff	44		9.24	2.14	10	0
Q6	Enough privacy at reception	259		8.90	2.38	10	0
Composite (Q4-Q6)		255		8.99	2.26	10	0

Note: Q1 and Q2 are categorical descriptors (previous SDEC attendance and referral source). Q3 is a binary filter item (“Were you taken to hospital by ambulance?”) and is not included in the composite score. Composite scores are calculated from Q4-Q6 only, with higher values indicating more positive arrival experiences. Percentages may not total 100% due to rounding.

Summary Statistics for Waiting Domain (Q7-Q10) & Composite Score

Question	n	Mean	SD	Median	IQR
Q7: Wait to speak to doctor/nurse	295	8.43	2.73	10.00	3.30
Q8: Wait to be examined	291	7.91	1.93	8.00	2.00
Q9: Information given on wait	233	4.36	4.69	0.00	10.00
Q10: Length of visit	236	6.59	1.89	6.70	3.30

Composite (Q7–Q9)	311	7.22	2.51	7.67	3.66

Note: Scores range from 0 to 10, with higher values indicating shorter waits. Composite scores reflect the mean of Q7-Q9. Question 10 (length of visit) is reported separately as a descriptive time indicator and is not included in the composite.

Summary Statistics for Doctors & Nurses Domain (Q11-Q17) & Composite Score

Question	n	Mean	SD	Median	IQR
Q11: Enough time to discuss health	289	9.43	1.85	10.00	0.00
Q12: Condition and treatment explained clearly	270	9.11	2.31	10.00	0.00
Q13: Felt listened to	282	9.75	1.17	10.00	0.00
Q14: Anxieties and fears discussed	203	8.79	2.66	10.00	0.00
Q15: Confidence and trust in staff	282	9.50	1.56	10.00	0.00
Q16: Not talked over (reverse scored)	282	0.83	2.62	0.00	0.00
Q17: Opportunity for family to speak to doctor	141	9.22	2.69	10.00	0.00
Composite (Q11–Q16)	295	7.82	1.42	8.33	0.83

Note: Scores range from 0 to 10, with higher values indicating more positive experiences. Question 16 was reverse scored so that higher values reflect respectful communication. The composite score includes Q11–Q16, capturing direct staff-patient interaction. Question 17 is reported separately due to its conditional relevance and third-party framing.

Summary Statistics for Your Care & Treatment Domain (Q18-Q22) & Composite Score

Question	n	Mean	SD	Median	IQR
Q18: Information about condition/treatment was appropriate	268	9.50	1.56	10.0	0.00
Q19: Enough privacy during examination/treatment	281	9.61	1.38	10.0	0.00
Q20: Able to get help from staff when needed	220	9.86	1.16	10.0	0.00
Q21: Not given conflicting information (reverse scored)	281	0.53	2.04	0.0	0.00
Q22: Involved in decisions as much as desired	276	8.93	2.42	10.0	0.00
Composite (Q18–Q22)	286	7.48	1.26	8	1

Note: Scores range from 0 to 10, with higher values indicating more positive experiences. Question 21 was reverse scored in the composite score so that higher values reflect consistent and non-conflicting information. The composite score includes Questions 18-22, capturing both informational clarity and patient involvement in care decisions.

Summary Statistics for Test Domain (Q24-Q26) & Composite Score

Question	n	Mean	SD	Median	IQR
Q24: Staff explained why tests were needed	261	8.91	2.60	10	0.00
Q25: Received test results before leaving	217	8.39	3.69	10	0.00
Q26: Results explained understandably	184	9.57	1.51	10	0.00
Composite (Q24–Q26)	214	8.73	2.31	10	1.67

Note: Question 23 was used to route respondents. Only those who had tests (Q23 = 1) were asked Q24-Q26. Question 26 was asked only if test results were received before leaving (Q25 = 10). The composite score includes Q24-Q26 for those who received results, and Q24-Q25 for those who did not. Scores range from 0 to 10, with higher values indicating more positive experiences.

Summary Statistics for Pain Domain (Q27-Q28)

Question	n	Mean	SD	Median	IQR
Q28: Staff did everything possible to help control pain	98	8.83	2.47	10	0

Note: Question 27 was used to route respondents. Only those who reported experiencing pain were asked Question 28. Scores range from 0 to 10, with higher values indicating that staff did everything possible to help control pain.

Summary Statistics for Environment & Facilities Domain (Q29-Q32) & Composite Score

Question	n	Mean	SD	Median	IQR
Q29: Cleanliness of SDEC	284	9.59	1.12	10	0.00
Q30: Cleanliness of toilets	211	9.15	1.69	10	0.00
Q31: Did not feel threatened	285	9.54	2.05	10	0.00
Q32: Suitable food and drinks available	261	9.54	2.10	10	0.00
Composite (Q29–Q32)	287	9.46	1.16	10	0.82

Note: Scores range from 0 to 10, with higher values indicating more positive experiences. Question 31 was framed negatively (“Did you feel threatened?”) and scored so that 10 reflects “No” and 0 reflects “Yes.” The composite score includes Questions 29-32, capturing cleanliness, safety, and access to food and drink.

Summary Statistics for Leaving SDEC Domain (Q35-Q41) & Composite Score

Question	n	Mean	SD	Median	IQR
Q35: Purpose of medication explained understandably	101	9.80	0.98	10	0
Q36: Side effects explained	87	6.72	4.37	10	10
Q37: Advice on resuming normal activities	128	6.60	4.30	10	10
Q38: Staff considered home situation	115	7.48	3.94	10	5
Q39: Advice on danger signals given	166	8.07	3.29	10	5
Q40: Information on who to contact with concerns	211	8.81	7.24	10	0
Q41: GP given necessary information	127	9.69	1.76	10	0
Composite (Q35-Q41)	216	8.19	4.28	10	3

Note: Question 33 was used to route respondents. Only those discharged from SDEC were asked subsequent questions. Question 34 determined whether new medicines were prescribed. If prescribed, respondents answered Q35-Q36, if not they skipped to Q37. The composite score includes Q35-Q41 for those with applicable responses. Scores range from 0 to 10, with higher values indicating more positive experiences.

Summary Statistics for Overall Experience Domain (Q42-Q44) & Composite Score

Question	n	Mean	SD	Median	IQR
Q42: Treated with dignity and respect	255	9.88	0.88	10	0
Q43: Overall experience (0–10 scale)	245	9.41	0.95	10	1
Q44: Saw information about how to complain	151	6.49	4.79	10	10
Composite (Q42–Q44)	260	9.00	1.72	10	1

Note: Scores range from 0 to 10, with higher values indicating more positive experiences. Question 42 asked whether respondents felt treated with respect and dignity. Question 43 used a 0-10 Likert scale to rate overall experience. Question 44 asked whether information about how to complain was available. The composite score includes all three items.
