# Supplementary material for: Patient experiences of acute medical same day emergency care: a mixed-methods cross-sectional survey across two hospitals in England
Source: BMJ Open. 2026 Oct 22;16(10):e124245. doi: 10.1136/bmjopen-2026-124245 (PMC13691836; doi:10.1136/bmjopen-2026-124245)
Supplement: Supplementary file 2 [file bmjopen-16-10-s003.pdf]

Additional File 3 Demographics by Site

**Variable**	**Category**	**Boston Site**<br>**Median (IQR) or n (%)**	**Lincoln Site**<br>**Median (IQR) or n (%)**
Age		61 (32)	58 (29.5)
	Valid n	*142*	*115*
Disability	No	61 (47.3%)	62 (56.9%)
	Prefer not to say	5 (3.9%)	2 (1.8%)
	Yes	63 (48.8%)	45 (41.3%)
	Valid n	*129*	*109*
Ethnicity	Other	15 (9.9%)	12 (10.1%)
	White British	137 (90.1%)	107 (89.9%)
	Valid n	*152*	*119*
Gender	Female	80 (56.3%)	66 (56.9%)
	Male	61 (43%)	49 (42.2%)
	Other	1 (0.7%)	1 (0.9%)
	Valid n	*142*	*116*
