# Supplementary material for: Patient experiences of acute medical same day emergency care: a mixed-methods cross-sectional survey across two hospitals in England
Source: BMJ Open. 2026 Oct 22;16(10):e124245. doi: 10.1136/bmjopen-2026-124245 (PMC13691836; doi:10.1136/bmjopen-2026-124245)
Supplement: Supplementary file 3 [file bmjopen-16-10-s004.pdf]

Additional File 4 Regression Model Outputs for Subgroup Analyses

Association Between Composite Scores and Site

Domain	Crude OR (CI)	Adjusted OR (CI)	Adjusted p	Chi Sq Statistic	Chi Sq p value	Cohen's h
Composite Arrival	0.69 (0.37-1.28)	0.78 (0.38-1.61)	0.5019	1.048	0.3059	0.143
Composite Waiting	0.71 (0.45-1.11)	0.78 (0.45-1.33)	0.3551	1.944	0.1632	0.173
Composite Doctors and Nurses	0.71 (0.36-1.40)	0.39 (0.14-1.02)	0.0618	0.676	0.4109	0.117
Composite Care and Treatment	1.26 (0.62-2.55)	1.00 (0.39-2.59)	0.9991	0.239	0.6253	-0.080
Composite Tests	0.75 (0.42-1.32)	0.77 (0.38-1.55)	0.4616	0.750	0.3864	0.124
Composite Pain	1.13 (0.43-2.96)	1.08 (0.35-3.57)	0.8911	0.001	0.9749	-0.056
Composite Environment	0.90 (0.41-1.99)	0.57 (0.17-1.80)	0.3397	0.001	0.9718	0.029
Composite Leaving SDEC	0.76 (0.45-1.29)	0.71 (0.39-1.29)	0.2648	0.782	0.3765	0.134
Composite Overall Experience	0.85 (0.47-1.53)	1.02 (0.53-1.97)	0.9628	0.155	0.6934	0.068

Note: Composite scores were binarised to indicate “positive experience” using a threshold of ≥8 on a 0-10 scale. Crude and adjusted odds ratios compare the odds of a positive composite score (≥8) between Lincoln and Boston (reference). Adjusted models include ethnicity, gender, age, and disability status. Chi-square statistics reflect Pearson’s test of independence for site and binary outcome. Cohen’s h quantifies the standardised difference in proportions between sites; values below 0.2 are considered negligible.

Association Between Composite Scores & Age Group (≤75 & >75)

Domain	Crude OR (CI)	Adj OR (CI)	Adj p	Chi Sq Statistic	Chi Sq p value	Cohen h
Composite Arrival	1.36 (0.56-3.86)	1.54 (0.61-4.46)	0.389	0.41	0.521	0.119
Composite Waiting	2.11 (1.07-4.31)	2.05 (1.01-4.3)	**0.0495**	4.58	**0.032**	0.369
Composite Doctors & Nurses	2.16 (0.59-13.93)	2.11 (0.54-14.05)	0.347	1.06	0.303	0.200
Composite Care and Treatment	2.23 (0.61-14.34)	2 (0.51-13.39)	0.381	1.16	0.282	0.211
Composite Tests	0.6 (0.27-1.42)	0.5 (0.2-1.26)	0.129	1.49	0.222	-0.219
Composite Pain	0.4 (0.09-2.14)	0.36 (0.07-2.04)	0.218	1.41	0.235	-0.404
Composite Environment	25440024.36 (0–NA)	-	unstable	3.19	0.074	0.554
Composite Leaving SDEC	1.11 (0.54–2.39)	1.17 (0.54–2.62)	0.696	0.08	0.779	0.051
Composite Overall Experience	0.91 (0.41–2.17)	0.89 (0.39–2.19)	0.788	0.05	0.818	-0.041

Note: Reference group is ≤ 75 years. Crude odds ratios were estimated using logistic regression with age group as the sole predictor. Adjusted odds ratios controlled for site, gender, ethnicity, and disability. Chi-squared tests compared binary domain outcomes between age groups using 2×2 contingency tables. Cohen's h was calculated manually using the arcsine transformation of group proportions; values of 0.2, 0.5, and 0.8 represent small, medium, and large effects respectively. "-" indicates unstable model estimates due to separation.

Association Between Composite Scores & Gender

Domain	Crude OR (CI)	Adj OR (CI)	Adj p	Chi sq Statistic	Chi sq p value	Cohen's h
Composite Arrival	0.92 (0.47-1.83)	1.07 (0.52-2.22)	0.8530	0.00	0.946	-0.032
Composite Waiting	0.71 (0.43-1.17)	0.64 (0.37-1.1)	0.1086	1.51	0.220	-0.171
Composite Doctors & Nurses	1.12 (0.5-2.59)	0.86 (0.33-2.31)	0.7677	0.00	0.946	0.03-
Composite Care and Treatment	2.1 (0.92-5.26)	1.56 (0.6-4.38)	0.3696	2.31	0.129	0.227
Composite Tests	1.73 (0.92-3.33)	1.93 (0.94-4.12)	0.0792	2.36	0.124	0.227
Composite Pain	1.51 (0.53-4.71)	1.5 (0.5-4.89)	0.4761	0.24	0.622	0.164
Composite Environment	2.48 (0.93-7.83)	2.86 (0.82-13.44)	0.1292	2.30	0.129	0.232
Composite Leaving SDEC	1.2 (0.69-2.12)	1.07 (0.59-1.97)	0.8199	0.26	0.612	0.089
Composite Overall Experience	0.96 (0.52-1.77)	0.86 (0.45-1.65)	0.6483	0.00	1.000	-0.019

Note: Adjusted odds ratios derived from logistic regression models controlling for site, age, ethnicity, and disability. Crude odds ratios reflect unadjusted gender comparisons. All comparisons are made with Female as the reference group. Chi-squared tests assess binary outcome distributions between Male and Female respondents. Cohen's h quantifies effect size for proportion differences, with values <0.2 considered small.

Association Between Composite Domains & Ethnicity Group (White British & Other)

Domain	Crude OR (CI)	Adj OR (CI)	Adj p	Chi sq stat	Chi sq or Fisher p value	Cohens h	Test
Composite Arrival	1.23 (0.38-5.53)	1.12 (0.33-5.13)	0.872		1	-0.0807	Fisher
Composite Waiting	0.38 (0.13-0.97)	0.39 (0.13-1.03)	0.0701	3.13	0.0770	0.4715	Chi-sq
Composite Doctors & Nurses	0.27 (0.09-0.91)	0.21 (0.06-0.78)	**0.0148**		**0.0321**	0.4532	Fisher
Composite Care and Treatment	0.21 (0.07-0.66)	0.2 (0.06-0.66)	**0.0067**		**0.0090**	0.5602	Fisher
Composite Tests	0.25 (0.1-0.65)	0.18 (0.06-0.48)	**<0.001**		**0.0046**	0.6309	Fisher
Composite Pain	1.4 (0.33-9.73)	1.26 (0.27-9.12)	0.791		1	-0.1334	Fisher
Composite Environment	0.15 (0.04-0.52)	0.18 (0.05-0.69)	**0.0087**		**0.0048**	0.5983	Fisher
Composite Leaving SDEC	0.91 (0.36-2.41)	0.79 (0.3-2.17)	0.641	0.00	1	0.0443	Chi-sq
Composite Overall Experience	0.42 (0.17-1.13)	0.37 (0.14-1.02)	**0.0473**		0.0942	0.3881	Fisher

Note: Crude and adjusted odds ratios compare the likelihood of a positive score (≥8) between White British and Other ethnicity groups, with White British as the reference category. Adjusted models include age, gender, site and disability. Chi-squared test used unless expected cell counts <5, in which case Fisher's exact test was applied. Cohen's h quantifies effect size between proportions. Test type is indicated in final column.

Association Between Composite Domains & Disability Status

Composite	Crude OR (CI)	Adj OR (CI)	Adj p	Chi sq stat	Chi sq p value	Cohen's h
Composite Arrival	0.68 (0.33-1.4)	0.63 (0.29-1.33)	0.2302	0.7428	0.3888	0.1510
Composite Waiting	1.03 (0.61-1.75)	0.84 (0.48-1.46)	0.5422	<0.0010	1	-0.0155
Composite Doctors & Nurses	0.47 (0.17-1.21)	0.31 (0.1-0.86)	**0.0303**	1.7483	0.1861	0.2133
Composite Care & Treatment	0.68 (0.26-1.72)	0.49 (0.17-1.32)	0.1645	0.3412	0.5592	0.1116
Composite Tests	0.69 (0.35-1.36)	0.6 (0.28-1.28)	0.1923	0.8072	0.3689	0.1513
Composite Pain	1.52 (0.52-4.66)	1.86 (0.6-6.25)	0.294	0.2386	0.6252	-0.1698
Composite Environment	1.44 (0.46-4.89)	0.97 (0.29-3.5)	0.9633	0.1115	0.7384	-0.0850
Composite Leaving SDEC	0.68 (0.37-1.23)	0.62 (0.33-1.16)	0.1388	1.2566	0.2623	0.1846
Composite Overall Experience	0.79 (0.41-1.51)	0.71 (0.36-1.41)	0.3317	0.3001	0.5838	0.0983

Note: Crude and adjusted odds ratios compare the likelihood of a positive score (≥8) between respondents who reported a disability (Yes) and those who did not (No), with No as the reference category. Adjusted models include site, age group, gender and ethnicity. Crude associations were assessed using Pearson’s chi-squared test. Cohen’s h quantifies effect size between proportions.
