# Supplementary material for: Patient experiences of acute medical same day emergency care: a mixed-methods cross-sectional survey across two hospitals in England
Source: BMJ Open. 2026 Oct 22;16(10):e124245. doi: 10.1136/bmjopen-2026-124245 (PMC13691836; doi:10.1136/bmjopen-2026-124245)
Supplement: Supplementary file 5 [file bmjopen-2026-124245.reviewer_comments.pdf]

PEER REVIEW HISTORY

BMJ Open publishes all reviews undertaken for accepted manuscripts. Reviewers are asked to complete a checklist review form and are provided with free text boxes to elaborate on their assessment. These free text comments are reproduced below.

ARTICLE DETAILS

Title (Provisional)

Patient experiences of acute medical Same Day Emergency Care: a mixed-methods cross-sectional survey across two hospitals in England

Authors

Dean, Sue; Blake, Holly; Barratt, Julian; Leonardi-Bee, Jo

VERSION 1 - REVIEW

**Reviewer**	**1**
**Name**	**Brandenburg, Caitlin**
**Affiliation**	**Goldcoast Health Service District, Office for Research Governance and Development**
**Date**	**16-Jul-2026**
**COI**	**None**

This an important and well written paper, however does need some editing before publication. Strengths of the paper are the mixed methods approach, handling of the quantitative methodological elements, and writing. Areas to be addressed are placing the study in the context of the international literature, clarity of the qualitative element, and some other minor clarifications to methods.

INTRODUCTION

From an international perspective, the SDEC model could be clearer in comparison to existing models. Describe the intended patients and process flow of the model a little more so comparisons to other contexts are clear (e.g. in Australian context, is this similar to an Ambulatory Care Unit, Short Stay Unit, Medical Assessment Unit, Minor Injuries and Illness Clinic?).

This is referred to as a novel model, but if I am understanding the model correctly, similar models have been established in Australia and perhaps other contexts. Please add further justification or information about why the model is novel, or if it is just novel in the UK.

Linked to the above, please provide more background on similar studies on patient experience in similar models, even if the model is novel it will strengthen the background to understand similar findings.

METHODS

"individuals who might require additional assistance to participate."- how was this element supported? Were the data collected by existing staff in-kind, in which case was it realistic they would have time to support the person to fill in the survey? Or were there paid Research Assistants or per-patient recruitment payments to offset this?

Please specify in methods that this was a paper rather than online survey- noted this is in limitations but needs to be clear earlier. Also if it was a physical paper survey, I'm unclear on use of "exited" in this sentence? "for Leaving SDEC, 257 where some participants exited the survey after being informed they could leave the unit."

Please also add somewhere the estimated time to complete the survey, likely available in previous research.

Please also add how surveys were returned. It was noted that "Leaving SDEC" was one of the categories, so presumably this would have been after discharge?

List in text the actual open ended questions asked for the reader to have context- "The final section invited free-text comments to complement the structured experience ratings."

"Free text comments were analysed thematically using NVivo (Version 15). A framework informed by the survey's conceptual structure was refined inductively through coding, generating themes relating to communication, interpersonal care, waiting and uncertainty, pain and comfort, coordination of care and overall satisfaction. These categories were then inductively refined and synthesised into three overarching themes." – the process of qualitative coding is very unclear. The term inductive is used, but coding seems to be deductive, coding comments to each of the survey's domains (Arrival, Waiting)? The remainder of the analysis does not appear in the results section- there is no reference to themes of "communication, interpersonal care, waiting and uncertainty, pain and comfort, coordination of care and overall satisfaction" or the "three overarching themes."

The "Other comments" section on the survey copy provided gives limited space for in-depth comments, so it is unclear whether there was enough data for a qualitative analysis. This is later touched upon in limitations. Please provide some more description around this, including the percent of respondents who did provide qualitative comments of some meaning (not just N/A or no comment), and the typical length of comments (e.g. mean 15 words, mostly 2-3 sentence under each question). It is also unclear if any consideration to data sufficiency/saturation was applied.

It is mentioned in methods that the SRQR was used, however elements are missing, and the CHERRIES checklist attached, but not the SRQR. However, I'm not sure the SRQR is needed or

can be applied, as this was a simple analysis of open ended survey text, rather than in depth qualitative study. Either delete reference to using the SRQR, or complete fully and attach.

I am skeptical that a true thematic analysis was applied (or even necessary) versus a qualitative content analysis. If so, please reference the specific thematic analysis approach you used along with clarifying the steps as above. Alternately, authors may find the ideas in this paper useful for methodologically defining the qualitative element of their study: Robinson, O. C. (2022). Conducting thematic analysis on brief texts: The structured tabular approach. Qualitative Psychology, 9(2), 194–208. https://doi.org/10.1037/qup0000189

Regardless of reporting guideline used, pertinent information is missing, such as who did the qualitative coding, what was their background, and how other authors contributed to the qualitative element of the analysis.

RESULTS

““From the minute we arrived… I was seen very quickly and 304 everyone treated me with care and kindness” [P292]”- this quote is used twice, please use only once or replace with another relevant quote.

“Regular patients…”- reads as a little informal/lacks clarity. Change to “Patients who had attended the SDEC regularly” or similar.

“…particularly regarding food quality and aspects of the” – missing the end of this sentence, appears to a be a typo

Under “Overall experience” the text “No negative comments were recorded in this phase.” Should be adjusted as overall experience isn’t really a phase the way “waiting” or “leaving” are.

DISCUSSION

In the discussion, results are described which are not covered in reported results: “Three overarching themes characterised positive patient experience: coordinated care, interpersonal communication, and environmental comfort. In contrast, uncertainty during waiting and variability in discharge information emerged as the main sources of frustration”

Regarding the findings around less positive responses from people of non-white ethnicities and with disabilities, were there any qualitative responses that helped explain this?

“Finally, the researcher’s insider position…” is described as a limitation, but the reader doesn’t know the researcher’s positionality. Please describe earlier in methods.

Is it also possible that a limitation was that the “leaving domain” was not able to be comprehensive, as many patient won’t fully know if there were given enough information until returning home and to daily life?

**Reviewer**	**2**
**Name**	**Truter, Piers**
**Affiliation**	**The University of Notre Dame Australia, School of Health Sciences**
**Date**	**24-Jul-2026**
**COI**	**None**

Thank you for the opportunity to review this manuscript on Same day emergency care. Contemporary universal health care jurisdictions must explore multiple points of system access for consumers, particularly where this links to care titrated to the specific needs of consumers. Further, systems cannot be run by counting activity alone and the experience of care is a known consumer priority.

Abstract

Line 32 – what is the variation that is being evaluated?

Line 34 – the study design listed here does not match the study design in the methods section. Consuder updating this.

Lines 40-42 – please align the research questions as outlined in the abstract with the research questions as stated at the end of the introduction

Line 54-55 – The thematic analysis is not stated in the results section.

Introduction:

The main focus of this manuscript is on Same Day Emergency Care (SDEC). It is not clear how this differs from an emergency department or outpatients. This context setting is essential for the manuscript. I suggest an additional heading in the methods where SDEC can be defined and the two facilities offering this service could be profiled. The profile should include scope and capacity elements. It's recommended to provide enough detail that a reader could consider the generalisability of the manuscript findings to their own health care setting.

Lines 91-95 – Is this sentence reflecting on ED activity? Is there data to justify this statement? Please define 'medical patient'. Please clarify where these patients are discharged from – ED? Hospital? MAU? This will give context to the suggestion that SDEC could reduce inpatient bed pressure. //This detail will enable a reader not familiar with the NHS context to understand the system issue that has led to the develop of SDEC.

Lines 103-4 – consider ending the sentence with the research question "To assess patient reported experiences of care processes and perceived quality with acute medical SDEC units in two hospitals in Lincolnshire". The remainder of the paragraph could move the to settings section of the manuscript.

Methods

Overall the methods section would be easier for a reader if it were organised under the following headings:

Study design

Setting

Participants (and in clearly stated inclusion and exclusion criteria)

The setting section may have inclusion criteria for SDEC – and this section inclusion criteria for the study

Recruitment (focus here on the process of recruitment)

Was this a paper or online survey?

Data collection (outlining the instruments and justification for using these)

Data analysis

Sampling (The specifics of how the final sample was established – this is a convenience sample?)

For instance Lines 113-14 – please move the justification for using the AEDQ to the Data collection sub-heading

Please provide more detail on the qualitative analysis – which team members were involved in coding / theme generation – if the PPIE involvement in here too.

It would be ideal to have a short reflexive statement on the research team members with particular focus on their relationship with the SDEC.

Results

Line 406 – this sentence does not appear to have an ending.

Discussion:

Lines 481-483 is there a reference to justify this statement of organisational features.

Line 482 – what is this comparison being made too? ED?

Line 523-525 – these themes do not seem to have been presented in the results section. The qualitative reporting has been on the 8 sections of patient experience. Please align the results and discussion.

VERSION 1 - AUTHOR RESPONSE

Reviewer: 1
Dr. Caitlin Brandenburg, Goldcoast Health Service District
Comments to the Author:

This an important and well written paper, however does need some editing before publication. Strengths of the paper are the mixed methods approach, handling of the quantitative methodological elements, and writing. Areas to be addressed are placing the study in the context of the international literature, clarity of the qualitative element, and some other minor clarifications to methods.

Thank you for the constructive feedback which has been addressed as per the points below.

INTRODUCTION
From an international perspective, the SDEC model could be clearer in comparison to existing models. Describe the intended patients and process flow of the model a little more so comparisons to other contexts are clear (e.g. in Australian context, is this similar to an Ambulatory Care Unit, Short Stay Unit, Medical Assessment Unit, Minor Injuries and Illness Clinic?).

This is referred to as a novel model, but if I am understanding the model correctly, similar models have been established in Australia and perhaps other contexts. Please add further justification or information about why the model is novel, or if it is just novel in the UK.

Thank you for this helpful observation. We agree that same-day and ambulatory approaches to acute care exist internationally, including in Australia. We have clarified this in the revised manuscript. However, the English acute medical SDEC model is novel in two specific respects:

1. Its national standardisation and mandated implementation across all acute hospitals in England, with defined service specifications, operating requirements, and performance expectations.
2. Its explicit design as a non-bedded, same-day acute medical assessment and treatment pathway, distinct from short-stay units, medical assessment units, and fast-track services used in other countries, which typically involve bed-based observation or lower-acuity presentations.

To reflect this, we have refined the text to state that the novelty of SDEC lies in its UK configuration, governance, and scale, rather than in the conceptual idea of same-day care. We also added brief reference to adjacent international models to improve clarity for readers.

Linked to the above, please provide more background on similar studies on patient experience in similar models, even if the model is novel it will strengthen the background to understand similar findings.

Thank you for this helpful comment. We explored international models, including those used in Australia, to identify comparable patient-experience studies. However, Australia

does not operate a non-bedded, same-day acute medical assessment model equivalent to SDEC. International services described as ambulatory care typically refer to outpatient clinics, ED fast-track areas, or bed-based short-stay and assessment units, which differ substantially from the English acute medical SDEC specification. As a result, genuinely comparable patient-experience studies are not available. We have clarified this in the revised manuscript and positioned SDEC's novelty in its nationally standardised, policy-mandated configuration within the England.

METHODS

"individuals who might require additional assistance to participate."- how was this element supported? Were the data collected by existing staff in-kind, in which case was it realistic they would have time to support the person to fill in the survey? Or were there paid Research Assistants or per-patient recruitment payments to offset this?

We have clarified in the Methods that assistance was limited to practical support (e.g., reading questions aloud or helping patients handle the paper survey) and did not involve interpretive guidance. Surveys were distributed by existing SDEC staff, and this level of assistance was feasible because SDEC patients are clinically stable and ambulatory by definition. No research assistants or per-patient recruitment payments were used.

Please specify in methods that this was a paper rather than online survey- noted this is in limitations but needs to be clear earlier. Also if it was a physical paper survey, I'm unclear on use of "exited" in this sentence? "for Leaving SDEC, 257 where some participants exited the survey after being informed they could leave the unit."

We now state explicitly that the survey was administered in paper format. The ambiguous wording around "exited" has been removed, and the return process is clarified (completed surveys were placed in a sealed collection box at the nurse's station).

Please also add somewhere the estimated time to complete the survey, likely available in previous research.

We have added that the survey took approximately 8-12 minutes to complete.

Please also add how surveys were returned. It was noted that "Leaving SDEC" was one of the categories, so presumably this would have been after discharge?

This is now included: surveys were returned to a collection box at the nurse's station. Participants were asked to comment on discharge instructions etc. prior to leaving the department.

List in text the actual open ended questions asked for the reader to have context- "The final section invited free-text comments to complement the structured experience ratings."

All three free-text questions are now listed in the Data Collection section.

"Free text comments were analysed thematically using NVivo (Version 15). A framework informed by the survey's conceptual structure was refined inductively through coding, generating themes relating to communication, interpersonal care, waiting and uncertainty, pain and comfort, coordination of care and overall satisfaction. These categories were then inductively refined and synthesised into three overarching themes." – the process of qualitative coding is very unclear. The term inductive is used, but coding seems to be deductive, coding comments to each of the survey's domains (Arrival, Waiting)? The remainder of the analysis does not appear in the results section- there is no reference to themes of "communication, interpersonal care, waiting and uncertainty, pain and comfort, coordination of care and overall satisfaction" or the "three overarching themes." The "Other comments" section on the survey copy provided gives limited space for in-depth comments, so it is unclear whether there was enough data for a qualitative analysis. This is later touched upon in limitations. Please provide some more description around this, including the percent of respondents who did provide qualitative comments of some meaning (not just N/A or no comment), and the typical length of comments (e.g. mean 15 words, mostly 2-3 sentence under each question). It is also unclear if any consideration to data sufficiency/saturation was applied.

We agree that the original description implied a full thematic analysis, which was not appropriate for brief free-text survey comments. We have therefore revised the Methods to describe the actual approach used: qualitative content analysis. Coding was guided by the AEDQ's patient-journey framework but began inductively through close reading of the data. Comments were not forced into predefined domains; instead, codes were refined to reflect the content of the responses, with many aligning naturally with the patient-journey stages. These descriptive categories are reported within each domain in the Results. This approach reflects the brevity of the comments (median 12 words) and avoids over-interpretation or claims of thematic saturation. We have also added further detail in the Results section to clarify the nature of the free-text data.

It is mentioned in methods that the SRQR was used, however elements are missing, and the CHERRIES checklist attached, but not the SRQR. However, I'm not sure the SRQR is needed or can be applied, as this was a simple analysis of open ended survey text, rather than in depth qualitative study. Either delete reference to using the SRQR, or complete fully and attach.

We have removed reference to SRQR, as it is not required for brief free-text analysis within a survey. Reporting is now guided solely by CROSS (the checklist that was uploaded).

I am skeptical that a true thematic analysis was applied (or even necessary) versus a qualitative content analysis. If so, please reference the specific thematic analysis approach you used along with clarifying the steps as above. Alternately, authors may find the ideas in this paper useful for methodologically defining the qualitative element of their study: Robinson, O. C. (2022). Conducting thematic analysis on brief texts: The structured tabular approach. Qualitative Psychology, 9(2), 194–208. https://doi.org/10.1037/qup0000189

We agree and have revised the description to qualitative content analysis. The analytic steps are now clearly described, including framework-guided coding, inductive refinement, and grouping codes into descriptive categories aligned with each stage of the patient journey.

Regardless of reporting guideline used, pertinent information is missing, such as who did the qualitative coding, what was their background, and how other authors contributed to the qualitative element of the analysis.

We have added detail on coder roles, supervisory oversight, and PPIE involvement. A reflexivity statement describing the lead researcher's background and positionality is also included.

RESULTS

""From the minute we arrived... I was seen very quickly and 304 everyone treated me with care and kindness" [P292]"- this quote is used twice, please use only once or replace with another relevant quote.
"Regular patients..."- reads as a little informal/lacks clarity. Change to "Patients who had attended the SDEC regularly" or similar.
"...particularly regarding food quality and aspects of the" – missing the end of this sentence, appears to a be a typo
Under "Overall experience" the text "No negative comments were recorded in this

phase." Should be adjusted as overall experience isn't really a phase the way "waiting" or "leaving" are.

Thank you, the manuscript has been updated accordingly.

DISCUSSION

In the discussion, results are described which are not covered in reported results: "Three overarching themes characterised positive patient experience: coordinated care, interpersonal communication, and environmental comfort. In contrast, uncertainty during waiting and variability in discharge information emerged as the main sources of frustration"

We have revised the Discussion to ensure it reflects the reported qualitative findings. References to "three overarching themes" have been replaced with wording consistent with qualitative content analysis, d*escribing* recurring positive patterns *related to coordinated care, interpersonal communication, and environmental comfort, and* sources of frustration *relating* to waiting and discharge information.

Regarding the findings around less positive responses from people of non-white ethnicities and with disabilities, were there any qualitative responses that helped explain this?

We have added clarification in the Discussion noting that the free-text comments did not provide subgroup-specific explanations for the less positive ratings among respondents from non-white ethnic backgrounds or those with disabilities, likely due to the brevity of comments.

"Finally, the researcher's insider position..." is described as a limitation, but the reader doesn't know the researcher's positionality. Please describe earlier in methods.

Thank you, positionality has been added in the methods section.

Is it also possible that a limitation was that the "leaving domain" was not able to be comprehensive, as many patient won't fully know if there were given enough information until returning home and to daily life?

We have added a limitation noting that patients may only recognise gaps in discharge information once they return home, meaning responses in the 'leaving' domain may not fully capture post-discharge informational needs.

Reviewer: 2
Dr. Piers Truter, The University of Notre Dame Australia, South Metropolitan Area Health Service Mount Pleasant
Comments to the Author:
Thank you for the opportunity to review this manuscript on Same day emergency care. Contemporary universal health care jurisdictions must explore multiple points of system access for consumers, particularly where this links to care titrated to the specific needs of consumers. Further, systems cannot be run by counting activity alone and the experience of care is a known consumer priority.

Thank you for your thoughtful comments. We have responded to each of the points in turn.

Abstract

Line 32 – what is the variation that is being evaluated?

Line 34 – the study design listed here does not match the study design in the methods section. Consider updating this.

Lines 40-42 – please align the research questions as outlined in the abstract with the research questions as stated at the end of the introduction

Line 54-55 – The thematic analysis is not stated in the results section.

Thank you for these helpful comments. We have revised the abstract to clarify the type of variation examined, updated the study design to match the Methods, aligned the outcomes with the research questions now stated explicitly in the Introduction, and amended the qualitative results description so it accurately reflects the content analysis reported in the Results.

Introduction:

The main focus of this manuscript is on Same Day Emergency Care (SDEC). It is not clear how this differs from an emergency department or outpatients. This context setting is essential for the manuscript. I suggest an additional heading in the methods where SDEC can be defined and the two facilities offering this service could be profiled. The profile should include scope and capacity elements. It's recommended to provide enough detail that a reader could consider the generalisability of the manuscript findings to their own health care setting.

Lines 91-95 – Is this sentence reflecting on ED activity? Is there data to justify this statement? Please define 'medical patient'. Please clarify where these patients are discharged from – ED? Hospital? MAU? This will give context to the suggestion that SDEC could reduce inpatient bed pressure. //This detail will enable a reader not familiar with the NHS context to understand the system issue that has led to the develop of SDEC.

Lines 103-4 – consider ending the sentence with the research question "To assess patient reported experiences of care processes and perceived quality with acute medical SDEC units in two hospitals in Lincolnshire". The remainder of the paragraph could move the to settings section of the manuscript.

Thank you for these helpful comments. We have strengthened the Introduction to provide clearer context for readers unfamiliar with the NHS. We have clarified how SDEC differs from both emergency departments and outpatient clinics by expanding the description of the pathway and contrasting it with related international models. We have also refined the sentence relating to 24-hour discharge to ensure it accurately reflects emergency admission activity. We now end the Introduction with the research question and have moved site-specific detail to a new "Study setting" subsection within the Methods.

Methods

Overall the methods section would be easier for a reader if it were organised under the following headings:

Study design

Setting

Participants (and in clearly stated inclusion and exclusion criteria)

The setting section may have inclusion criteria for SDEC – and this section inclusion criteria for the study

Recruitment (focus here on the process of recruitment)

Was this a paper or online survey?

Data collection (outlining the instruments and justification for using these)

Data analysis

Sampling (The specifics of how the final sample was established – this is a convenience sample?)

For instance Lines 113-14 – please move the justification for using the AEDQ to the Data collection sub-heading

We thank you for these helpful suggestions. The Methods section has been reorganised and a Study Setting section added. We have clarified the distinction between SDEC service criteria (described in the Study Setting section) and study inclusion criteria (now clearly stated in the Sampling and Recruitment section). Inclusion criteria specify that participants were adults (≥18 years), clinically stable, able to provide informed consent, and sufficiently well to complete a short paper survey during their SDEC visit. Recruitment procedures are now described in detail under Sampling and Recruitment. We explicitly state that the survey was administered in paper format, distributed by SDEC staff during the patient's visit. The justification for using the AEDQ has been moved to the Data Collection section, where the instrument is described.

Please provide more detail on the qualitative analysis – which team members were involved in coding / theme generation – if the PPIE involvement in here too.

The qualitative analysis description has been expanded. We now specify that the lead researcher conducted initial coding in NVivo, academic supervisors reviewed and contributed to the refinement of coding categories, and a PPIE representative reviewed the qualitative interpretation to ensure patient perspectives were appropriately reflected.

It would be ideal to have a short reflexive statement on the research team members with particular focus on their relationship with the SDEC.

A reflexive statement has been added at the end of the Data Analysis section. This outlines the lead researcher's dual role as a PhD candidate and Advanced Nurse Practitioner working within SDEC and describes how academic supervisors (independent of participating sites) and a PPIE representative contributed to analytic oversight.

Results

Line 406 – this sentence does not appear to have an ending.

Thank you, this typo has been corrected.

Discussion:

Lines 481-483 is there a reference to justify this statement of organisational features.

We have added a reference to support this statement.

Line 482 – what is this comparison being made too? ED?

We agree that the comparator was not explicitly stated in the original wording. The sentence has now been revised to clarify that these organisational features are described in contrast to Emergency Departments (ED), consistent with NHS England's SDEC Service Specification.

Line 523-525 – these themes do not seem to have been presented in the results section. The qualitative reporting has been on the 8 sections of patient experience. Please align the results and discussion.

The Discussion has now been revised to align with the reported qualitative findings, which are structured around the patient-journey domains. The text has been updated to refer to recurring patterns within these domains rather than theme

---

VERSION 2 - REVIEW

Reviewer 1

Name Brandenburg, Caitlin

Affiliation Goldcoast Health Service District, Office for Research Governance and Development

Date 06-Aug-2026

COI

Thank you to the authors for changes to further strengthen this important paper. It is now suitable for publication.

Reviewer 2

Name Truter, Piers

Affiliation The University of Notre Dame Australia, School of Health Sciences

Date 16-Aug-2026

COI

Thank you for addressing the issues raised in the first review.
