# Supplementary material for: Patient experiences of acute medical same day emergency care: a mixed-methods cross-sectional survey across two hospitals in England
Source: BMJ Open. 2026 Oct 22;16(10):e124245. doi: 10.1136/bmjopen-2026-124245 (PMC13691836; doi:10.1136/bmjopen-2026-124245)
Supplement: Supplementary file 6 [file bmjopen-16-10-s001.docx]

Additional File 1: The SDEC Survey

Same Day Emergency Care (SDEC)

Department Questionnaire

### What is the survey about?

This survey is about your **most recent** visit to the Same Day Emergency Care department.

### Who should complete the questionnaire?

The questions should be answered by the patient. If that person needs help to complete the questionnaire, the answers should be given from his/her point of view – not the point of view of the person who is helping.

### Completing the questionnaire

For most questions, please tick clearly inside one box using a black or blue pen. For some questions you will be instructed that you may tick more than one box.

Sometimes you will find the box you have ticked has an instruction to go to another question. By following the instructions carefully, you will miss out questions that do not apply to you.

Don’t worry if you make a mistake; simply cross out the mistake and put a tick in the correct box.

Please do not write your name or address anywhere on the questionnaire.

### Questions or help?

If you have any questions, please ask the nursing staff or contact Sue Dean, Same Day Emergency Care, Pilgrim Hospital, Sibsey Road, Boston, PE21 9QS.

Sue.dean2@ulh.nhs.uk

**Taking part in this survey is voluntary.**

**Your answers will be treated in confidence.**

**ARRIVAL AT THE SAME DAY EMERGENCY CARE (SDEC) DEPARTMENT**

Please remember, this questionnaire is about your **most recent** visit to the Same Day Emergency Care department,

1. Before your most recent visit to SDEC, had you previously been to SDEC about **the same condition** or something related to it?

1 🞏 Yes, within the previous week

2 🞏 Yes, between one week and one month earlier

3 🞏 Yes, more than a month earlier

4 🞏 No

5 🞏 Don’t know / can’t remember

2. Who advised you to go to the SDEC Department? **(Tick ONE only – if more than one option applies, tick the MAIN source of advice)**

1 🞏 The ambulance service

2 🞏 A doctor or nurse at a walk-in center or minor injuries unit

3 🞏 A GP out of hours service

4 🞏 A GP from my local surgery

5 🞏 Some other health professional (e.g. NHS Direct nurse)

6 🞏 Somebody else (e.g. friend, relative, colleague)

7 🞏 The A&E department

8 🞏 Don’t know / can’t remember

## Travelling by ambulance

3. Were you taken to the hospital in an ambulance?

1 🞏 Yes	🡺 **Go to 4**

2 🞏 No	🡺 **Go to 6**

4. How well do you think the ambulance service and SDEC staff worked together?

1 🞏 Very well

2 🞏 Fairly well

3 🞏 Not very well

4 🞏 Not at all well

5 🞏 Don’t know / can’t remember

5. Once you arrived at hospital, how long did you wait with the ambulance crew before your care was handed over to the SDEC staff?

1 🞏 I did not have to wait

2 🞏 Up to 15 minutes

3 🞏 16 – 30 minutes

4 🞏 31 – 60 minutes

5 🞏 More than 1 hour but no more than 2 hours

6 🞏 More than 2 hours

7 🞏 Don’t know / can’t remember

## Reception

6. Were you given enough privacy when discussing your condition with the receptionist?

1 🞏 Yes, definitely

2 🞏 Yes, to some extent

3 🞏 No

4 🞏 I did not discuss my condition with a receptionist

# WAITING

7. How long did you wait before you **first spoke** to a nurse or doctor?

1 🞏 0 -15 minutes

2 🞏 16 - 30 minutes

3 🞏 31- 60 minutes

4 🞏 More than 60 minutes

5 🞏 Don’t know / can’t remember

8. From the time you first arrived at the SDEC Department, how long did you wait **before being examined** by a doctor or nurse?

1 🞏 I did not have to wait	🡺 **Go to 10**

2 🞏 1-30 minutes	🡺 **Go to** **9**

3 🞏 31-60 minutes	🡺 **Go to** **9**

4 🞏 More than 1 hour but no more than 2 hours

🡺 **Go to** **9**

5 🞏 More than 2 hours but no more than 4 hours

🡺 **Go to** **9**

6 🞏 More than 4 hours	🡺 **Go to** **9**

7 🞏 Can’t remember	🡺 **Go to** **9**

8 🞏 I did not see a doctor or a nurse

🡺 **Go to** **10**

9. Were you told **how long** you would have to wait to be examined?

1 🞏 Yes, but the wait was **shorter**

2 🞏 Yes, and I had to wait about as long as I was told

3 🞏 Yes, but the wait was **longer**

4 🞏 No, I was not told

5 🞏 Don’t know / can’t remember

10. Overall, how long did your visit to the **SDEC Department** last?

1 🞏 Up to 1 hour

2 🞏 More than 1 hour but no more than 2 hours

3 🞏 More than 2 hours but no more than 4 hours

4 🞏 More than 4 hours but no more than 6 hours

5 🞏 More than 6 hours but no more than 8 hours

6 🞏 More than 8 hours but no more than 12 hours

7 🞏 More than 12 hours but no more than 24 hours

8 🞏 More than 24 hours

9 🞏 Can’t remember

# DOCTORS AND NURSES

11. Did you have **enough time** to discuss your health or medical problem with the doctor or nurse?

1 🞏 Yes, definitely	🡺 **Go to** **12**

2 🞏 Yes, to some extent	🡺 **Go to** **12**

3 🞏 No	🡺 **Go to** **12**

4 🞏 I did not see a doctor or nurse 🡺 **Go to** **18**

12. While you were in the SDEC Department, did a doctor, advanced practitioner or nurse explain your condition and treatment in a way you could understand?

1 🞏 Yes, completely

2 🞏 Yes, to some extent

3 🞏 No

4 🞏 I did not need an explanation

13. Did the doctors, advanced practitioners and nurses listen to what you had to say?

1 🞏 Yes, definitely

2 🞏 Yes, to some extent

3 🞏 No

14. If you had any anxieties or fears about your condition or treatment, did a doctor, advanced practitioner or nurse discuss them with you?

1 🞏 Yes, completely

2 🞏 Yes, to some extent

3 🞏 No

4 🞏 I did not have anxieties or fears

15. Did you have confidence and trust in the doctors, advanced practitioners and nurses examining and treating you?

1 🞏 Yes, definitely

2 🞏 Yes, to some extent

3 🞏 No

16. Did doctors, advanced practitioners or nurses talk in front of you as if you weren’t there?

1 🞏 Yes, definitely

2 🞏 Yes, to some extent

3 🞏 No

17. If your family or someone else close to you wanted to talk to a doctor or advanced practitioner, did they have enough opportunity to do so?

1 🞏 Yes, definitely

2 🞏 Yes, to some extent

3 🞏 No

4 🞏 No family or friends were involved

5 🞏 My family or friends did not want or need information

6 🞏 I did not want my family or friends to talk to a doctor

# YOUR CARE AND TREATMENT

18. While you were in the SDEC Department, how much information about your condition or treatment was given to **you**?

1 🞏 Not enough

2 🞏 Right amount

3 🞏 Too much

4 🞏 I was not given any information about my condition or treatment

19. Were you given enough privacy when **being examined or treated**?

1 🞏 Yes, definitely

2 🞏 Yes, to some extent

3 🞏 No

20. If **you** needed attention, were you able to get a member of staff to help you?

1 🞏 Yes, always

2 🞏 Yes, sometimes

3 🞏 No, I could not find a member of staff to help me

4 🞏 A member of staff was with me all the time

5 🞏 I did not need attention

21. Sometimes in a hospital, a member of staff will say one thing and another will say something quite different. Did this happen to you in the SDEC Department?

1 🞏 Yes, definitely

2 🞏 Yes, to some extent

3 🞏 No

22. Were you involved as much as you wanted to be in decisions about your care and treatment?

1 🞏 Yes, definitely

2 🞏 Yes, to some extent

3 🞏 No

4 🞏 I was not well enough to be involved in decisions about my care

# TESTS

23. Did you have any tests (such as x-rays, scans or blood tests) when you visited the SDEC Department?

1 🞏 Yes	🡺 **Go to 24**

2 🞏 No	🡺 **Go to** **27**

24. Did a member of staff explain **why you needed**

these test(s) in a way you could understand?

1 🞏 Yes, completely

2 🞏 Yes, to some extent

3 🞏 No

25. Before you left the SDEC Department, did you get the **results** of your tests?

1 🞏 Yes	🡺 **Go to 26**

2 🞏 No	🡺 **Go to 27**

3 🞏 I was told that the results of the tests would be given to me at a later date	🡺 **Go to 27**

4 🞏 Don’t know / can’t remember	🡺 **Go to 27**

26. Did a member of staff explain the **results of the tests** in a way you could understand?

1 🞏 Yes, definitely

2 🞏 Yes, to some extent

3 🞏 No

4 🞏 Not sure / can’t remember

# PAIN

27. Were you in any pain while you were in the SDEC Department?

1 🞏 Yes	🡺 **Go to 28**

2 🞏 No	🡺 **Go to** **29**

28. Do you think the hospital staff did everything they could to help control your pain?

1 🞏 Yes, definitely

2 🞏 Yes, to some extent

3 🞏 No

4 🞏 Can’t say / don’t know

# HOSPITAL ENVIRONMENT AND FACILITIES

29. In your opinion, how clean was the SDEC Department?

1 🞏 Very clean

2 🞏 Fairly clean

3 🞏 Not very clean

4 🞏 Not at all clean

5 🞏 Can’t say

30. How clean were the toilets in the SDEC Department?

1 🞏 Very clean

2 🞏 Fairly clean

3 🞏 Not very clean

4 🞏 Not at all clean

5 🞏 I did not use a toilet

31. While you were in the SDEC Department, did you feel threatened by other patients or visitors?

1 🞏 Yes, definitely

2 🞏 Yes, to some extent

3 🞏 No

32. Were you able to get suitable food or drinks when you were in the SDEC Department?

1 🞏 Yes

2 🞏 No

3 🞏 I was told not to eat or drink

4 🞏 I did not know if I was allowed to eat or drink

5 🞏 I did not want anything to eat or drink

# LEAVING THE SDEC DEPARTMENT

33. What happened at the end of your visit to the SDEC Department?

1 🞏 I was admitted to the same hospital

🡺 **Go to 42**

2 🞏 I was transferred to a different hospital or to a nursing home	🡺 **Go to 42**

3 🞏 I went home	🡺 **Go to** **34**

4 🞏 I went to stay with a friend or relative

🡺 **Go to** **34**

5 🞏 I went to stay somewhere else 🡺 **Go to** **34**

**Medications (e.g. medicines, tablets, ointments)**

34. Before you left the SDEC department, were any **new** medications prescribed for you?

1 🞏 Yes	🡺 **Go to** **35**

2 🞏 No	🡺 **Go to** **37**

35. Did a member of staff explain **the purpose** of the medications you were to take at home in a way you could understand?

1 🞏 Yes, completely

2 🞏 Yes, to some extent

3 🞏 No

4 🞏 I did not need an explanation

36. Did	a	member	of	staff	tell	you	about

**medication side effects** to watch for?

1 🞏 Yes, completely

2 🞏 Yes, to some extent

3 🞏 No

4 🞏 I did not need this type of information

## Information

37. Did a member of staff tell you when you could **resume your usual activities**, such as when to go back to work or drive a car?

1 🞏 Yes, definitely

2 🞏 Yes, to some extent

3 🞏 No

4 🞏 I did not need this type of information

38. Did hospital staff take your **family or home situation** into account when you were leaving the SDEC Department?

1 🞏 Yes, completely

2 🞏 Yes, to some extent

3 🞏 No

4 🞏 It was not necessary

5 🞏 Don’t know / can’t remember

39. Did a member of staff tell you about what **danger signals** regarding your illness or treatment to watch for after you went home?

1 🞏 Yes, completely

2 🞏 Yes, to some extent

3 🞏 No

4 🞏 I did not need this type of information

40. Did hospital staff tell you **who to contact** if you were worried about your condition or treatment after you left the SDEC Department?

1 🞏 Yes

2 🞏 No

3 🞏 Don’t know / can’t remember

41. As far as you know, was your GP given all the necessary information about the treatment or advice that you received in the SDEC Department?

1 🞏 Yes

2 🞏 No

3 🞏 Don’t know

4 🞏 No information was needed

# OVERALL

42. Overall, did you feel you were treated with respect and dignity while you were in the SDEC Department?

1 🞏 Yes, all of the time

2 🞏 Yes, some of the time

3 🞏 No

43. Overall...

| I had a very poor  experience | I had a very good  experience |
| --- | --- |

0	1	2	3	4	5	6	7	8	9	10

44. While in the SDEC Department, did you ever see any posters or leaflets explaining how to complain about the care you received?

1 🞏 Yes

2 🞏 No

**49.** Do you have any of the following long-standing conditions? (TICK ALL THAT APPLY)

1 🞏 Deafness or severe hearing impairment

🡺 **Go to** **50**

2 🞏 Blindness or partially sighted

3 🞏 Don’t know/ can’t remember

# ABOUT YOU

45. Are you male or female?

1 🞏 Male	🞏 Female

2 🞏 Other	🞏 Prefer not to say

46. What was your **year** of birth?

| **(Please write in)** e.g. | 1 | 9 | 3 | 4 |
| --- | --- | --- | --- | --- |

47. What is your religion?

1 🞏 No religion

2 🞏 Buddhist

3 🞏 Christian (including Church of England, Catholic, Protestant, and other Christian denominations)

4 🞏 Hindu

5 🞏 Jewish

6 🞏 Muslim

7 🞏 Sikh

8 🞏 Other

9 🞏 I would prefer not to say

48. Which of the following best describes how you think of yourself?

1 🞏 Heterosexual / straight

2 🞏 Gay / lesbian

3 🞏 Bisexual

4 🞏 Other

5 🞏 I would prefer not to say🡺 **Go to** **50**

3 🞏 A long-standing physical condition

🡺 **Go to** **50**

4 🞏 A learning disability	🡺 **Go to** **50**

5 🞏 A mental health condition	🡺 **Go to** **50**

6 🞏 A long-standing illness, such as cancer, HIV, diabetes, chronic heart disease, or epilepsy

🡺 **Go to** **50**

7 🞏 No, I do not have a long-standing condition

🡺 **Go to 51**

50. Does this condition(s) cause you difficulty with any of the following? (TICK ALL THAT APPLY)

1 🞏 Everyday activities that people your age can usually do

2 🞏 At work, in education or training

3 🞏 Access to buildings, streets or vehicles

4 🞏 Reading or writing

5 🞏 People’s attitudes to you because of your condition

6 🞏 Communicating, mixing with others or socialising

7 🞏 Any other activity

8 🞏 No difficulty with any of these

51. How many times (including this one) have you visited an SDEC department **as a patient** in the last 12 months?

1 🞏 This was the only time

2 🞏 2 – 3 times

3 🞏 4 – 5 times

4 🞏 6 or more times

5 🞏 Don’t know / can’t remember

52. To which of these ethnic groups would you say you belong? **(Tick ONE only)**

#### a. WHITE

1 🞏	English/Welsh/Scottish/Northern Irish/ British

2 🞏	Irish

3 🞏	Gypsy or Irish Traveler

4 🞏	Any other White background,

**write in...**

#### b. MIXED / MULTIPLE ETHNIC GROUPS

5 🞏	White and Black Caribbean

6 🞏	White and Black African

7 🞏	White and Asian

8 🞏	Any other Mixed/multiple ethnic background, **write in...**

#### c. ASIAN / ASIAN BRITISH

9 🞏	Indian

10🞏	Pakistani 11🞏	Bangladeshi 12🞏	Chinese

13🞏	Any other Asian background,

**write in...**

#### d. BLACK / AFRICAN / CARIBBEAN / BLACK BRITISH

14 🞏	African

15 🞏	Caribbean

16 🞏	Any other Black / African / Caribbean background, **write in...**

#### e. OTHER ETHNIC GROUP

17 🞏	Arab

18 🞏	Any other ethnic group, **write in...**

# ANY OTHER COMMENTS

If there is anything else you would like to tell us about your experiences in the SDEC Department, please do so here.

Was there anything particularly good about your visit to the SDEC Department?

Was there anything that could have been improved?

Any other comments?

#### Were you seen in Boston Lincoln

#### THANK YOU VERY MUCH FOR YOUR HELP

**Please check that you answered all the questions that apply to you.**

**Please hand this anonymous survey to nursing staff as you leave.**
